# Development of New Antimycobacterial Sulfonyl Hydrazones and 4-Methyl-1,2,3-thiadiazole-Based Hydrazone Derivatives

**DOI:** 10.3390/antibiotics11050562

**Published:** 2022-04-22

**Authors:** Violina T. Angelova, Tania Pencheva, Nikolay Vassilev, Elena K-Yovkova, Rositsa Mihaylova, Boris Petrov, Violeta Valcheva

**Affiliations:** 1 Department of Chemistry, Faculty of Pharmacy, Medical University, 1431 Sofia, Bulgaria; bobi.stoyanov@abv.bg; 2 Department of QSAR and Molecular Modeling, Institute of Biophysics and Biomedical Engineering, Bulgarian Academy of Sciences, 1113 Sofia, Bulgaria; tania.pencheva@biomed.bas.bg; 3 Laboratory “Nuclear Magnetic Resonance”, Institute of Organic Chemistry with Centre of Phytochemistry, Bulgarian Academy of Sciences, 1113 Sofia, Bulgaria; nikolay.vassilev@orgchm.bas.bg; 4 Faculty of Computer Systems and Technologies, Technical University, 1756 Sofia, Bulgaria; epi_ka@abv.bg; 5 Laboratory “Drug Metabolism and Drug Toxicity”, Department “Pharmacology, Pharmacotherapy and Toxicology”, Faculty of Pharmacy, Medical University, 1431 Sofia, Bulgaria; rositsa.a.mihaylova@gmail.com; 6 Laboratory of Molecular Biology of Mycobacteria, Department of Infectious Microbiology, The Stephan Angeloff Institute of Microbiology, Bulgarian Academy of Sciences, 1113 Sofia, Bulgaria

**Keywords:** antimycobacterial activity, ADME/Tox predictions, cytotoxicity, hydrazide-hydrazone derivatives, sulfonyl hydrazone derivatives, molecular docking

## Abstract

Fifteen 4-methyl-1,2,3-thiadiazole-based hydrazone derivatives **3a**–**d** and sulfonyl hydrazones **5a**–**k** were synthesized. They were characterized by ^1^H-NMR, ^13^C NMR, and HRMS. *Mycobacterium tuberculosis* strain H37Rv was used to assess their antimycobacterial activity. All compounds demonstrated significant minimum inhibitory concentrations (MIC) from 0.07 to 0.32 µM, comparable to those of isoniazid. The cytotoxicity was evaluated using the standard MTT-dye reduction test against human embryonic kidney cells HEK-293T and mouse fibroblast cell line CCL-1. 4-Hydroxy-3-methoxyphenyl substituted 1,2,3-thiadiazole-based hydrazone derivative **3d** demonstrated the highest antimycobacterial activity (MIC = 0.0730 µM) and minimal associated cytotoxicity against two normal cell lines (selectivity index SI = 3516, HEK-293, and SI = 2979, CCL-1). The next in order were sulfonyl hydrazones **5g** and **5k** with MIC 0.0763 and 0.0716 µM, respectively, which demonstrated comparable minimal cytotoxicity. All compounds were subjected to ADME/Tox computational predictions, which showed that all compounds corresponded to Lipinski’s Ro5, and none were at risk of toxicity. The suitable scores of molecular docking performed on two crystallographic structures of enoyl-ACP reductase (InhA) provide promising insight into possible interaction with the InhA receptor. The 4-methyl-1,2,3-thiadiazole-based hydrazone derivatives and sulfonyl hydrazones proved to be new classes of lead compounds having the potential of novel candidate antituberculosis drugs.

## 1. Introduction

Despite the significant progress in the development of new drugs and vaccines against tuberculosis (TB), new treatment regimens and health control programs did not prevent the spread of the disease. Multiple factors, such as the global economic crisis, human migration, alcohol and drug addiction, and the spread of HIV infection, seriously impact the TB incidence and lead to the emergence of *Mycobacterium tuberculosis* strains resistant to the widely used anti-TB drugs [1,2,3,4]. In addition, the World Health Organization anticipates that under the conditions of the COVID-19 pandemic, TB victims are exposed to higher health risks [1,5,6]. The risks can be reduced by developing new anti-TB compounds that shorten treatment time and, at low therapeutic doses, have a specific effect on multidrug-resistant strains. The search for new drug candidates with specific chemical, microbiological, and pharmacological characteristics has become increasingly urgent.

**Figure 1:**
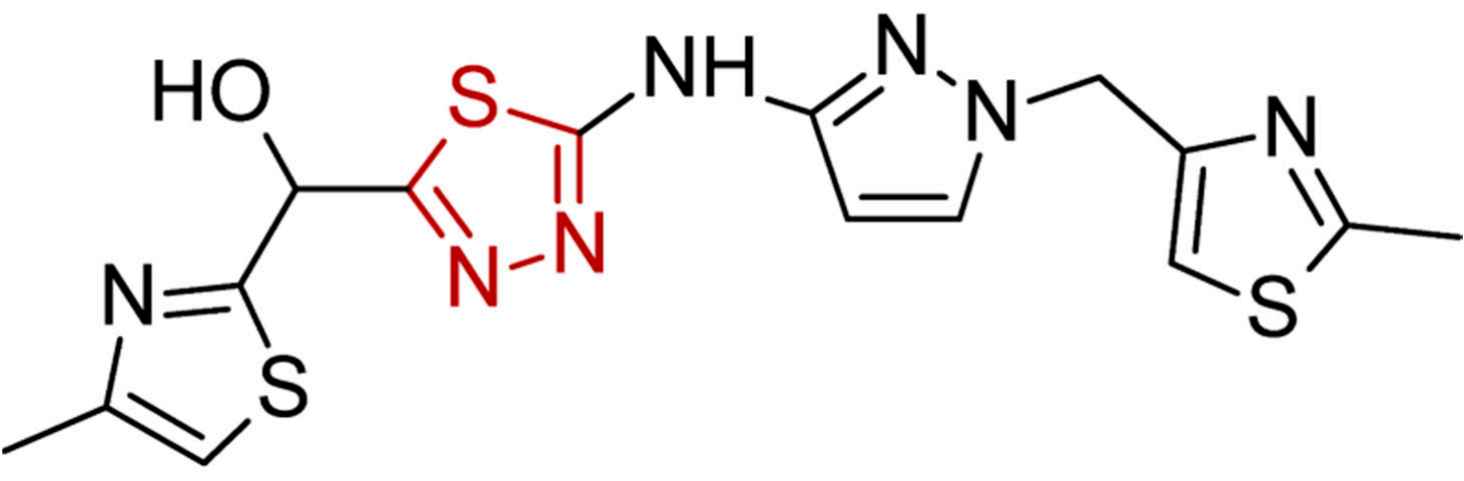
The chemical structure of GSK-693-novel direct Mtb InhA inhibitor.

Isoniazid (INH) remains important as the first-line anti-TB drug. INH is a prodrug, and its activity depends on bioactivation by KatG to form an isonicotinoyl-NAD (INH-NAD) adduct and subsequent inhibition of InhA [7]. InhA catalyzes the NADH-dependent reduction in long-chain trans-2-enoyl-acyl carrier proteins (ACPs). The KatG activation dependency of INH has been one of the main clinical weaknesses associated with INH use due to KatG mutations leading to INH resistance [8]. Furthermore, Hegde et al. [7] concluded that the observed SAR of INH largely reflects the restricted substrate specificity for acyl-NAD adduct formation rather than the initial KatG oxidation and subsequent InhA inhibition. Indeed, pyridomycin, or natural isoniazid, reported by Hartkoorn et al. [9], is a natural product that also inhibits InhA and whose binding closely overlaps with the INH-NAD adduct. Thus, identifying inhibitors that directly bind to InhA without the requirement for activation by KatG (direct InhA inhibitors) may be a valid strategy to overcome INH resistance [10]. For example, GSK-693 (Figure 1) is a novel direct reversible InhA inhibitor of *M. tuberculosis* that binds to the active site and is currently being studied as a potential substitute for isoniazid in current TB treatment regimens [11]. Researchers from GlaxoSmithKline plc. (GSK), under the TB Alliance sponsorship, carried out a screen against InhA using the GSK compound collection and identified the thiadiazole series as the most promising antitubercular drug family [12].

On the other hand, many hydrazide-hydrazone derivatives with antimycobacterial activity have been developed [11,13,14,15,16,17]. The replacement of the isonicotinic acid with a variety of substituted aromatic fragments exhibits a higher minimal inhibitory concentration (MIC) of the new derivatives against *M. tuberculosis* H37Rv. The hydrazones were shown to be three to four times more potent than INH [14,18,19,20]. Introducing the hydrazone moiety to the structure of new drug candidates as well as marketed antimycobacterial agents such as isoniazid [21], ciprofloxacin [22], and pyrazinamide [23] is a rational and frequently used approach to obtain novel anti-TB molecules with reduced toxicity. In our previous work, we performed *M. tuberculosis* H37Rv growth inhibition assays of a series of hydrazone-containing melatonin analogs. 2,3-Thiadiazole-containing hydrazone with a *p*-methoxyindol scaffold had excellent antimycobacterial activity against the reference strain *M. tuberculosis* H37Rv (MIC value 0.39 μM), low cytotoxicity, and no toxic effects when administered by oral or intraperitoneal routes to experimental animals (selectivity index SI > 1979, LD_50_ > 2000 mg/kg b.w.), which revealed its suitability for further exploration. As a result, unlike INH, the compound did not affect the urine and serum hematological and biochemical parameters compared to the control mice. The new compound did not significantly influence the MDA quantity and maintained its level near the control values compared with the INH-treated animals. At the higher doses, 200 and 400 mg/kg, its level remained 47% higher than that in the INH-treated animals. Encouraged by our previous experience and literature data about thiadiazole derivatives [24], we carried out further experiments to develop novel hydrazone derivatives with a 4-methyl-1,2,3-thiadiazole substituent as potential antitubercular agents with improved drug properties.

Alternatively, *N*-arylsulfonyl hydrazone derivatives were developed as antimycobacterial agents [15,25,26,27] to overcome the resistance mechanisms generated by some bacterial species [28,29]. Sulphonyl hydrazones are considered to be a promising scaffold for antitubercular drug discovery, which prompts further studies on their mechanism of action to completely validate InhA as the main molecular target. Additionally, the sulfonyl hydrazones were chosen because of their antimicrobial [27,28,30,31,32,33,34,35,36], anticancer [37,38,39,40,41,42,43,44,45,46], antiviral [47], and antifungal [48,49,50,51] properties, and inhibition of metabolic enzymes, in particular, carbonic anhydrase (CA) isoenzymes [52]. Besides sulfonyl, hydrazones have antidepressant properties [53,54], insecticidal activity [55,56], α-glycosidase, and acetylcholinesterase inhibitory properties [45,57,58,59], and the ability to inhibit some other enzymes [60,61,62].

These investigations encouraged us to develop new compounds containing hydrazones with a 4-methyl-1,2,3-thiadiazole scaffold and *N*-substituted sulfonyl hydrazones. For this purpose, tests on the antimycobacterial activity, cytotoxicity, and in silico ADME properties of these compounds were performed combined with a molecular docking study to determine the mechanism of action.

## 2. Results

### 2.1. Chemistry

**Scheme 1:**
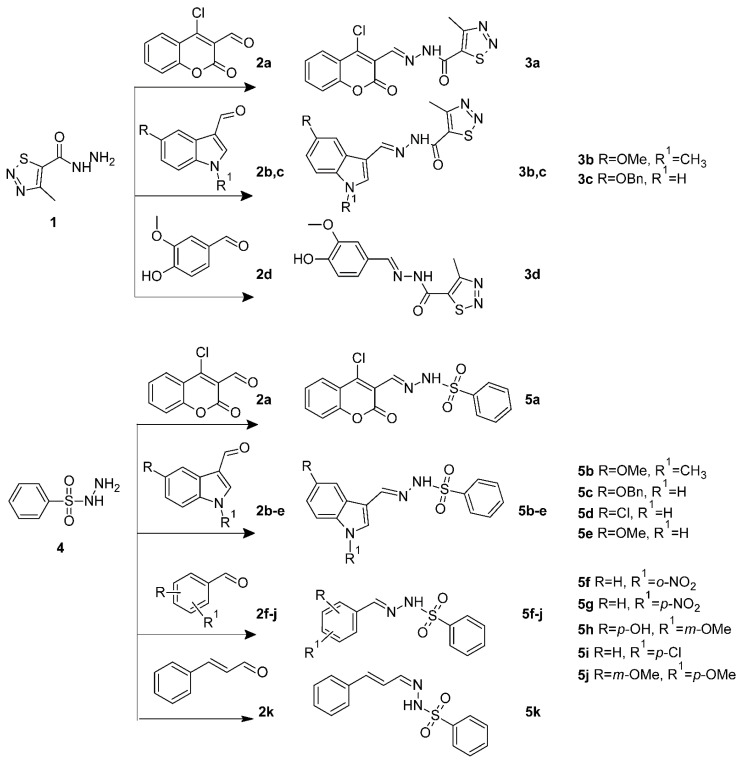
Synthesis of hydrazones with a 4-methyl-1,2,3-thiadiazole scaffold (**3a**–**d**) and *N*-substituted sulfonyl hydrazones (**5a**–**k**)**.**

The addition of protecting groups to the terminal nitrogen of the molecule by the formation of hydrazide-hydrazones is a widely studied strategy for lessening the limitations of INH. In this way, it is possible to avoid reactions at this site and, consequently, the inactivation and generation of toxic metabolites. Hydrazones **3a**–**d** were prepared by the condensation reaction of a 4-methyl-1,2,3-thiadiazole-5-carbohydrazide **1** and aldehydes **2a**–**d**, at a molar ratio of 1:1, in abs. ethanol for 1–2 h. The exploited synthetic strategy to develop the target derivatives is presented in Scheme 1. Among the newly synthesized compounds, compound **3a** was reported previously [19]. The ^1^H-NMR spectra of compounds **3b**–**d** showed single signals corresponding to resonances of azomethine protons (CH=N) at 8.08–8.38 ppm. The hydrazide/hydrazone N/H protons were observed at 11.73–12.25 ppm. The ^13^C-NMR spectra of **5a**–**g** exhibited resonances arising from azomethine (C=N) at 143.04 to 146.16 and hydrazide/hydrazone (C=O) carbons at 162.57–163.19, respectively. The following NMR experiments: 2D COSY, DEPT-135, 2D NOESY, 2D HSQC and 2D HMBC, were used for the precise structure elucidation of all new compounds (see Supplementary data). Sulfonyl hydrazones **5a**–**k** were synthesized by procedures similar to those shown in Scheme 1. Subsequently, we investigated the use of an acid catalyst. Treating the reaction mixture with a catalytic amount of *p*-toluenesulfonic acid (PTSA; 10 mol%) in refluxing ethanol afforded a suitable yield of the corresponding sulfonyl hydrazones after half the reaction time. Surprisingly, this reaction condition provided the desired product in almost quantitative yields. The structures of all compounds were confirmed by ^1^H NMR, ^13^C NMR, and HRMS spectroscopic data. The ^1^H-NMR spectra of **5a**–**k** had single signals corresponding to resonances of azomethine protons (CH=N) at 8.08–8.38 ppm. The hydrazide/hydrazone N/H protons were observed at 10.93–12.01 ppm. The ^13^C-NMR spectra of **5a**–**k** exhibited resonances arising from azomethine (C=N) at 144.62 to 149.48, respectively (see Supplementary Materials).

### 2.2. M. Tuberculosis Growth Inhibition and Cytotoxic Activity of Novel Compounds against Normal Cell Lines

Among the 15 compounds synthesized, **3d**, **5g**, and **5k** were found to be the most active compounds at MIC less than 0.1 µM and were similarly active as INH against *M. tuberculosis* H37Rv (Table 1). Two different series of compounds were compared: hydrazone derivatives with 4-methyl-1,2,3-thiadiazole fragment **3a**–**d** and sulfonyl hydrazones **5a**–**k**. Improved activity with MICs in the micro- to submicromolar concentration range was observed by varying the substituents in sulfonyl hydrazone derivatives **5a**–**k**. Concerning the first family of hydrazones, compound **3d** with a 4-methyl-1,2,3-thiadiazole heterocyclic fragment and a 4-hydroxy-3-methoxy-substituted phenyl ring exhibited the highest activity. Noteworthy introduction of a sulfonyl hydrazone fragment in the second series and the lack of a thiadiazole ring, despite one of the 4-hydroxy-3-methoxy-substituted benzene rings in compound **5h**, resulted in lower MIC values. Again, compound **5c** bearing a sulfonyl hydrazone fragment exhibited a two-fold higher MIC than compound **3c**, which was a hydrazone derivative with a thiadiazole ring. As seen, two compounds of the first family, **3c** and **3d**, showed the best inhibitory activity, compared to the compounds in both series, and MIC below 0.08 μM. The other two compounds, **3a** and **3b**, showed an opposite tendency compared to the compounds from the second series, **5a** and **5b** (MIC < 0.4 µM). These results proved that replacing the hydrazone group with sulfonyl hydrazone functionality was not always a perspective modification to obtain more active antitubercular agents. Despite this fact, the sulfonyl hydrazone derivative prepared from cinnamic aldehyde **5k** had the best activity (MIC = 0.07 µM) among the second family **5a**–**k**, commensurate with **3d** (MIC = 0.07 µM) from the first family. To assess the safety profile of the compounds, we used Mosmann’s MTT assay to evaluate their cytotoxicity against two normal cell lines, HEK-293 (human embryonic kidney 293 cells) and CCL-1 (mouse fibroblast cell line). The results of the study (calculated IC_50_ values) are presented in Table 1. The compound with the most potent antimycobacterial activity (MIC of 0.07 µM) **3d** demonstrated negligible cytotoxicity to the non-malignant human embryonic kidney cells HEK-29 and mouse fibroblast cells CCL-1 (corresponding IC_50_ values of 256.7 and 217.5 µM, respectively), in vitro test systems commonly used for verifying biocompatibility. We also calculated the selectivity indices (SI) as the ratio of IC_50_ to MIC, and values higher than 10 were indicative of acceptable toxicity. Table 1 reports SI values higher than 10 for all tested compounds. Compounds **3b**–**d**, **5g**, and **5k** demonstrated the most prominent biocompatibility and selectivity for the tubercular bacilli with SIs > 1000. The most potent compound, **3d**, demonstrated very low toxicity against the non-malignant human embryonic kidney cells HEK-29 and mouse fibroblast cells CCL-1 and high selectivity index values (SI = 3516 and 2979, respectively for both screened cell lines), followed by **3c** (SI = 2242 and SI = 4093) and **5k** (SI = 3380 and SI = 2216, respectively), indicating that they are selective for *M. tuberculosis* infection.

A comparison of **3d** with the previously mentioned similar scaffold, a compound with a 4-methyl-1,2,3-thiadiazole moiety and a 5-metoxindole scaffold [20] (which activity is two-fold more potent than isoniazid and four-fold higher than ethambutol) revealed the importance of the 1,2,3-thiadiazole moiety in the connecting side chain for future research in the development of novel antitubercular agents. Additionally, the oral administration of the compound with a 4-methyl-1,2,3-thiadiazole moiety and 5-metoxindole scaffold [63], at the highest dose of 2000 mg/kg b.w. resulted in no mortalities or evidence of adverse effects, implying that the compound is non-toxic. Thus, comparable to isoniazid, the 4-methyl-1,2,3-thiadiazole-based hydrazone derivatives **3b**–**d** and **5k**, which are small, non-toxic synthetic molecules, can be readily prepared and are excellent drug candidates.

### 2.3. ADME/Tox Screening Results

The newly synthesized compounds were subjected to an in silico ADME screening study. The specific properties were compared to the available drugs used for treating TB (Table 2), namely, molecular weight (MW), topological polar surface area (TPSA), hydrogen bond acceptors (HBA), hydrogen bond donors (HBD), Moriguchi’s LogP (MLogP), and water solubility.

All synthesized compounds had molecular weights in the qualifying range between 160 and 480 g/mol. The higher MW corresponded to poor bioavailability, poor fraction absorption, and higher bond fraction [64]. The TPSA values between 67 and 125 Å^2^ indicated suitable absorption through the cell membrane. Most of the compounds appeared to be moderately soluble except the compounds with coumarin scaffold **3a** and **5a**, as well as 5-substituted indole derivatives **5c** and **5d** that showed poor solubility in water. It is worth noting that the most active compound, **3d**, was predicted to have aqueous solubility similar to ethambutol and isoniazid. The lipophilicity (LogP) value is a key property for predicting the oral liability of drug molecules. A drug targeting the central nervous system (CNS) should have a LogP value of approximately 2; for oral and intestinal absorption, the ideal value is 1.35–1.8, while a drug intended for sub-lingual absorption should have a logP value > 5. Thus, the range of LogP from 0 to 5 is acceptable for an effective drug. In this study, the compounds’ values ranged from 0.03 to 2.61, which is the adequate limit for a drug to penetrate the bio-membrane. The **3a**–**d** compounds have lower lipophilicity than **5a**–**d**, where the hydrazone fragment was replaced with the sulfonyl hydrazone fragment. The most active compound, **3d**, with a predicted LogP of 0.03, demonstrated characteristics similar to isoniazid and ethambutol (LogP −0.47, LogP 0.18, respectively). According to Moriguchi et al. [65], all other compounds have higher lipophilicity. We support the idea [66] that lipophilic compounds can easily penetrate through the cell wall of *M. tuberculosis*; thus, lipophilic molecules have great therapeutic value as future antitubercular agents. Cinnamaldehyde sulfonyl hydrazone derivative **5k** showed LogP values of 2.52 (Table 2) and activity similar to that of vanillin aldehyde hydrazone derivative **3d** (Table 1).

The drug-likeness was evaluated using Lipinski’s “Rule of Five”, Ghose fitter, and Veber’s constraints [67], and our synthesized compounds were shown to have a suitable ADMET profile to be considered oral drug candidates (Table 3). According to the pharmacokinetic properties (Table 3), all compounds showed high gastrointestinal absorption, most of them had no BBB permeability (except **5i** and **5k**), and only **5f** and **5g** were predicted to be P-gp substrates. The inhibition of the cytochrome P450 (CYP) family enzyme isoforms could lead to unwanted effects or a higher risk of hepatotoxicity [67,68,69,70,71]. In order to avoid such a situation, the synthesized compounds were computationally evaluated according to the inhibition of several CYP450 isoforms: CYP1A2, CYP2C19, CYP2C9, CYP2D6, and CYP3A4. The in silico molecular screening revealed that two of the compounds, 3d and 5h, were predicted to be non-inhibitors of the CYP450 isoforms, **5a** could inhibit only CYP2C19, **5f**, and **5g** were predicted to be inhibitors only of CYP1A2. The other compounds were predicted to exhibit inhibition properties over two or more of the enzyme isoforms.

Then, the toxic potential of the new compounds due to their chemical structure was predicted by the web service ProTox-II assessment (https://tox-new.charite.de/protox_II, accessed on 21 April 2022). The evaluation scheme included classification into several levels/classes of toxicity [72] for each of the following endpoints: oral toxicity (acute rodent toxicity), organ toxicity (hepatotoxicity), and toxicological endpoints (such as carcinotoxicity, immunotoxicity, mutagenicity, and cytotoxicity). The classification was based on the predicted LD_50_ value, given in mg/kg, according to the globally harmonized system of classification of labeling the chemicals (GHS). As shown in Table 4, most of the components belonged to class IV (300 < LD_50_ ≤ 2000), two (**3c, 3d**) were predicted in class V (2000 < LD_50_ ≤ 5000) and the other two (**3a, 5k**) were predicted in class III (50 < LD_50_ ≤ 300). According to some studies, INH was confirmed to be in class III, and EMB was confirmed to be in class IV. All of the new components from classes V and IV were taken into consideration in our studies. In addition, the balanced probability of the predicted toxicities was estimated at a certain confidence rate for each of the endpoints. The results are summarized in Table 4, where the compounds were classified into two classes: active and inactive, with the corresponding probability value. Probability values above 70% were taken into serious consideration for both active (A) and inactive (I) cases.

The hepatotoxicity simulation tests revealed that all compounds from group **5a**–**k** were predicted to be non-hepatotoxic or hepatotoxic inactive, but only **5g** showed hepatotoxic activity with a low probability score of 0.52. Surprisingly, the components from groups **3a**–**d** were predicted to be hepatotoxic active but with lower probability values of 0.61, 0.51, 0.63, and 0.60, respectively. Nevertheless, these values remained lower or comparable to the corresponding probabilities of EMB and INH. The prediction tests of some genotoxicity end-points-immunotoxicity and cytotoxicity determined all of the components to be both non-immunotoxic and non-cytotoxic with high levels of probability. Only **3b** was predicted to be immunotoxicity active with a relatively high probability value (0.78). The carcinotoxicity prediction study demonstrated that most of the compounds were inactive (**3a**–**c, 5a**–**e, 5i**), and the rest were active at low probability rates. The simulations at the mutagenicity endpoint showed different behavior: compounds **3a**–**d** were predicted to be mutagenically active at a low probability-0.52, 0.54, 0.55, and 0.56, respectively, while other compounds were revealed to be mutagenically inactive.

### 2.4. Molecular Docking

Docking simulations were carried out in Molecular Operating Environment (MOE, https://www.chemcomp.com/MOE-Molecular_Operating_Environment.htm, version 2016.08, accessed on 18 April 2022) with the following two X-ray crystallographic structures of *M. tuberculosis* enoyl reductase (InhA):

(1) The crystal structure of *M. tuberculosis* InhA complexed with 5-hexyl-2-(2-methylphenoxy)phenol (TCU) with the co-factor nicotinamide adenine dinucleotide (NAD^+^) was extracted from the Protein Data Bank (http://www.rcsb.org/ (accessed on 20 April 2022), PDB ID 2X22);
(2) The crystal structure of *M. tuberculosis* InhA complexed with (3S)-1-cyclohexyl-N-(3,5-dichlorophenyl)-5-oxopyrrolidine-3-carboxamide (ligand ID 641, further denoted as 641), also with a co-factor NAD^+^, extracted from PDB (PDB ID 4TZK).

Dogan et al. [73] have reported some promising results when investigating hydrazone-containing thiadiazoles as InhA inhibitors, which makes the crystal structure 4TZK the first choice for molecular docking studies of the compounds considered here. Their interest in the crystal structure 2X22 has been provoked by the results reported by Menendez et al. [74].

Table 5 lists the docking scores for the synthesized compounds in both structures 2X22 and 4TZK. The obtained docking scores of all the compounds in both enzyme structures were found to be satisfactory, in the range of −12.36 to −10.02 in 2X22 (Column 2X22, E-score 1 in Table 5) and from −14.67 to −11.10 in 4TZK (Column 4TZK, E-score 1 in Table 5). The results presented in Table 5 show that the two most active compounds (lowest MIC), namely, **5k** and **3d** appeared as the two top-ranked compounds after docking in 2X22. The third most active compound, **5g**, reached the fourth-best rank after docking in 2X22, while **5e** showed the third-best rank. The docking scores were found to be −12.36 (**5k)**, −12.19 (**3d**), −12.03 (**5e**), and −11.98 (**5g**) (Table 5). The docking procedure in 4TZK distinguished **5e** as the top-ranked compound, with a docking score of −14.67, followed by **5k** (with a docking score of −12.83) and **5g** (with a docking score of −12.80), with both being the most active and the third most active compounds. The second active compound, **3d**, achieved a docking score of −12.38, placing it at the sixth position in the docking ranking. In both enzyme structures, the reference compound isoniazid recorded the worst results, with docking scores of −9.18 in 2X22 and −8.51 in 4TZK.

**Figure 2:**
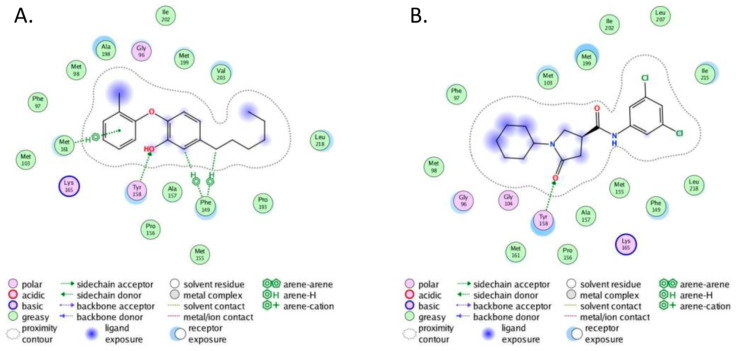
Interaction diagrams of the ligand-binding domains of *M. tuberculosis* InhA with: (**A**) 5-hexyl-2-(2-methylphenoxy)phenol (TCU) (PDB ID 2X22) and (**B**) (3s)-1-cyclohexyl-N-(3,5-dichlorophenyl)-5-oxopyrrolidine-3-carboxamide (641) (PDB ID 4TZK).

The protein–ligand interactions (PLI) diagrams of the co-crystalized ligands of *M. tuberculosis* InhA in the ligand-binding domains of both receptors, 2X22 and 4TZK, were obtained using the “Ligand Interactions” tool of MOE at the maximum distance of 4.5 Å between the heavy atoms of the ligands and receptors. Figure 2 presents the PLI of the ligands of both receptors for TCU (2X22, Figure 2A) and 641 (4TZK, Figure 2B).

Docking studies performed on isoniazid derivatives demonstrated that the hydrogen bond interactions with Tyr158 and Ile194 were critical for binding InhA, which is in agreement with previous results on Ser94, Gly96, Lys165, and Ile194 [15,72,75,76,77].

Phe149, Tyr158, and Met161 were involved in protein–ligand interactions, forming arene-H interactions (Phe149 and Met161), and H-bond (Tyr158). Residues Gly96, pro193, Ala198, Met199, val201, and Leu218 were at receptor exposure, very close to the ligand, but still not at the binding distance (Figure 2A). Only Tyr158 was involved in protein–ligand interactions, forming H-bond. Residues Gly96, Met103, Phe149, Met199, Ile215, and Leu218 are at receptor exposure, very close to the ligand, but still not at a binding distance (Figure 2B).

**Figure 3:**
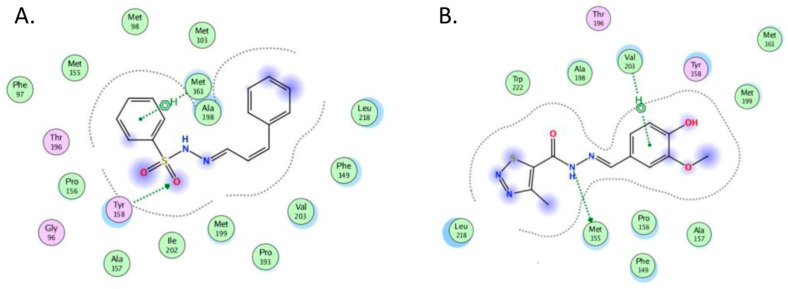
Interaction diagrams of the ligand-binding domain of *M. tuberculosis* InhA (2X22) with the most active compounds and top-scored poses of (**A**) **5k** and (**B**) **3d**.

Figure 3 presents the PLI diagrams of the most active compounds **5k** and **3d**, which were the top-scored compounds after docking in the receptor 2X22.

Figure 3A (with a legend equal to that presented in Figure 2) shows that the most active and the top-scored compound **5k** demonstrated the reproduction of two out of three interactions as presented in Figure 2A, namely with Tyr158 and Met161, while the second active and second top-scored compound **3d** did not repeat the specific interaction with Tyr158, but produced two newly appeared interactions with Met155 (H-bond) and Val203 (arene-H interaction).

**Figure 4:**
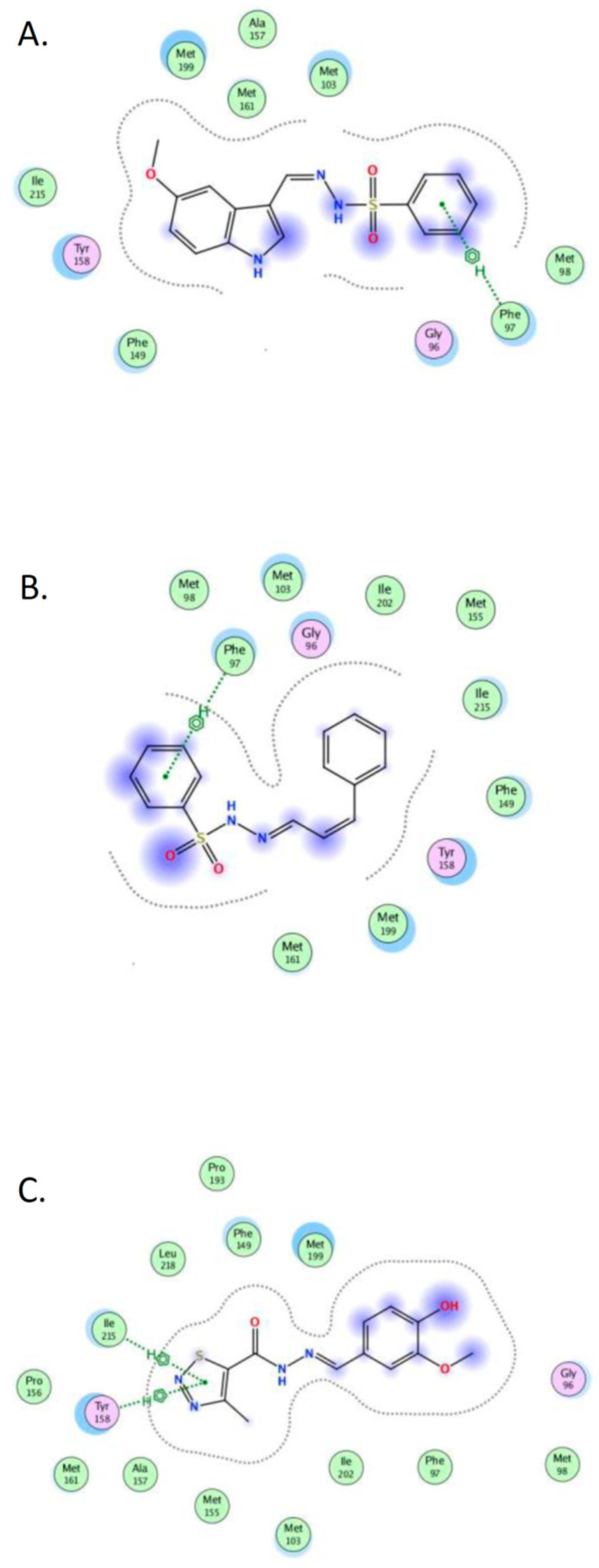
Interaction diagrams of the ligand-binding domains of *M. tuberculosis* InhA (4TZK) with the top-scored compounds of (**A**) **5e**; (**B**) **5k**; and (**C**) **3d**.

Figure 4 presents the PLI diagrams of the top-scored compounds after docking in the receptor 4TZK, namely, **5e** and **5k**, and one of the most active compounds, **3d**.

The top-scored compound **5e** demonstrated only one newly appeared interaction with Phe97 (arene-H interaction) (Figure 4A). Most of the mentioned important residues were still very close but did not exhibit any strong interaction. The second-ranked and one of the most active compounds, **5k**, presented in Figure 4B, also demonstrated only one newly appeared interaction with Phe97 (arene-H interaction). As in the case of compound **5e**, most of the mentioned important residues were still very close. Noticeably, the catalytic residue Tyr158 did not form any H-bonds with the inhibitor. Thus, **5e** and **5k** turned out to be representative of a class of inhibitors with no need for a conserved network of interaction with Tyr158 for potency. One of the most active compounds, **3d** (Figure 4C), repeated the PLI interaction with Tyr158, as shown in Figure 2B, demonstrating a new interaction with Ile 215 (both arene-H interactions). We suggest that the main differences in the binding mode rely on the hydrophobicity of the two series compounds-hydrazone derivatives with 4-methyl-1,2,3-thiadiazole fragment **3a**–**d** and sulfonyl hydrazones **5a**–**k**.

**Figure 5:**
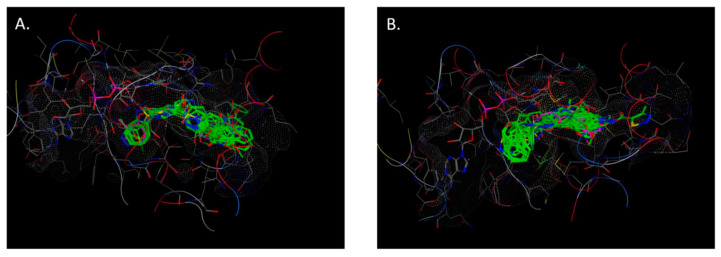
Docking conformations of all synthesized compounds (in green), the ligands TCU (**A**) (in magenta) and 641 (**B**) (in magenta), and the corresponding Connolly surface.

All synthesized compounds, docked in the ligand-binding domains of *M. tuberculosis* InhA, are presented in Figure 5 in the receptor 2X22 (Figure 5A) and the receptor 4TZK (Figure 5B) with a Connolly surface. All synthesized compounds occupied the same binding site as the respective ligands, forming clusters that fit well in the ligand-binding domain of *M. tuberculosis* InhA.

**Figure 6:**
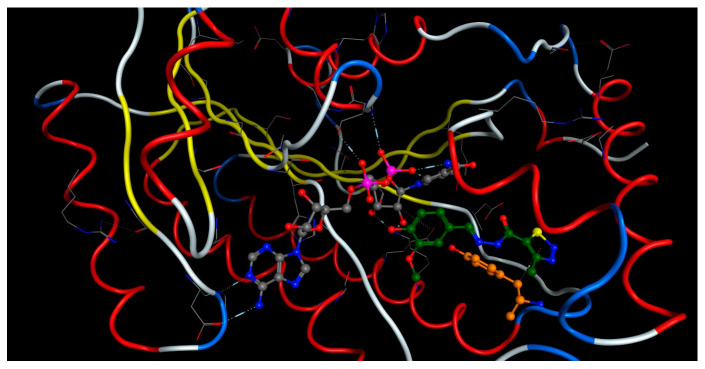
Interactions of **3d** (green) in the ligand-binding domains of *M. tuberculosis* InhA (4TZK) with NAD+ (in gray) and Tyr158 (in orange).

Figure 6 presents a closer view of the interactions of one of the most active compounds **3d** in the ligand-binding domain of *M. tuberculosis* InhA (4TZK), demonstrating the noticed scores above PLI with Tyr158, as well as the H-bond with NAD^+^.

The interactions of **3d** (in green) in the ligand-binding domains of *M. tuberculosis* InhA (4TZK) with NAD+ (in gray) and Tyr158 (in orange) presented in Figure 6 support the hypothesis that **3d** might be considered an inhibitor binding directly to InhA without the requirement for activation by KatG. Taken together, our data suggest that **3d** potentially targets InhA and that its mechanism of action is independent of KatG activation. The direct mode of binding to InhA and circumventing the main isoniazid resistance mechanisms would lead these compounds to be active against MDR-TB clinical isolates. The further planned investigations on the in vitro inhibition of InhA will shed light on whether the sulfonyl hydrazones and 4-methyl-1,2,3-thiadiazole-containing hydrazone derivatives bind InhA and would help to understand the protein-compound molecular interactions in vivo.

## 3. Materials and Methods

### 3.1. Chemistry

The melting points were determined using a Buchi 535 apparatus and melting point meter M5000 apparatus. All nuclear magnetic resonance (NMR) experiments were carried out on a Bruker Avance spectrometer 600 MHz at 20 °C in deuterated dimethyl sulfoxide (DMSO-d6) as a solvent and tetramethylsilane (TMS) as the internal standard. The precise assignment of the ^1^H and ^13^C NMR spectra was accomplished by measurement of two-dimensional (2D) homonuclear correlation (correlation spectroscopy (COSY)), DEPT-135, and 2D inverse-detected heteronuclear (C-H) correlations (heteronuclear single-quantum correlation spectroscopy (HMQC) and heteronuclear multiple bond correlation spectroscopy (HMBC)). Mass spectra were measured on a Q Exactive Plus mass spectrometer (ThermoFisher Scientific) equipped with a heated electrospray ionization (HESI-II) probe (Thermo Scientific). All chemicals used for the synthesis were commercial products and used without further purification.

#### 3.1.1. General Procedure for the Synthesis of **3a**–**d**

To a solution of 4-methyl-1,2,3-thiadiazole-5-carbohydrazide **1** (2.0 mmol) in absolute (abs.) ethanol, a stirred solution of appropriate carbaldehydes **2a**–**d** (2.0 mmol) in abs. ethanol was added. The solution was refluxed for 1–3 h. The solid product formed was collected by filtration and recrystallized with ethanol.

*N’-[(E)-(4-chloro-2-oxo-2H-1-benzopyran-3-yl)methylidene]-4-methyl-1,2,3-thiadiazole-5-carbohydrazide*, **3a** [19] Yield: 77%; m.p. 252–253 °C. HRMS (ESI) *m*/*z*: calcd: [M+H]^+^ 337.165902. Found: [M+H]^+^ 337.16517.
*N’-[(E)-(5-methoxy-1-methyl-1H-indol-3-yl)methylidene]-4-methyl-1,2,3-thiadiazole-5-carbohydrazide*, **3b** Yellow solid. Yield: 77%; m.p. 191–192 °C. ^1^H NMR (600 MHz, DMSO-d_6_) δ 11.98 (s, 1H, NH), 8.35 (s, 1H, CH=N), 7.90 (s, 1H, H-2), 7.67 (d, *J* = 2.4 Hz, 1H, H-4), 7.45 (d, *J* = 8.9 Hz, 1H, H-7), 6.93 (dd, *J* = 2.4, 8.9 Hz, 1H, H-6), 3.83 (s, 3H, OCH_3_), 3.82 (s, 3H, NCH_3_), 2.96 (s, 3H, CH_3_). ^13^C NMR (151 MHz, DMSO-d_6_) δ 162.63 (C=O), 158.95 (C-4′), 155.03 (C-5), 143.04 (CH=N), 136.41 (C-5′), 135.94 (C-2), 132.67 (C-7a), 124.53 (C-3a), 112.97 (C-6), 111.64 (C-7), 109.22 (C-3), 102.31 (C-4), 55.45 (OCH_3_), 33.14 (NCH_3_), 14.83 (CH_3_). HRMS (ESI) *m*/*z*: calcd: [M+H]^+^ 330.10192. Found: [M+H]^+^ 330.1009.
*N’-((E)-[5-(benzyloxy)-1H-indol-3-yl]methylidene)-4-methyl-1,2,3-thiadiazole-5-carbohydrazide*, **3c** Yellow solid. Yield: 76%; m.p. 229–230 °C. ^1^H NMR (600 MHz, DMSO-d_6_) δ 12.02 (s, 1H, NH-indol), 11.73 (s, 1H, NH), 8.38 (s, 1H, CH=N), 7.92 (d, *J* = 2.3 Hz, 1H, H-2), 7.80 (d, *J* = 2.4 Hz, 1H, H-4), 7.49–7.47 (m, 2H, *o*-Ar), 7.42–7.39 (m, 3H, *m*-Ar and H-7), 7.33 (tt, *J* = 1.5, 7.4 Hz, 1H, *o*-Ar), 6.97 (dd, *J* = 2.4, 8.8 Hz, 1H, H-6), 5.15 (s, 2H, CH_2_), 2.97 (s, 3H, CH_3_). ^13^C NMR (151 MHz, DMSO-d_6_) δ 162.57 (C=O), 159.01 (C-5‘), 153.80 (C-5), 143.59 (CH=N), 137.27 (*i*-Ar), 136.61 (C-4′), 132.86 (C-2), 132.16 (C-7a), 128.40 (*m*-Ar), 128.10 (*o*-Ar), 127.75 (*p*-Ar), 124.00 (C-3), 113.64 (C-6), 113.12 (C-7), 110.50 (C3a), 103.69 (C-4), 69.84 (CH_2_), 14.79 (CH_3_). HRMS (ESI) *m*/*z*: calcd: [M+H]^+^ 392.117571. Found: [M+H]^+^ 392.1166
*N’-[(E)-(4-hydroxy-3-methoxyphenyl)methylidene]-4-methyl-1,2,3-thiadiazole-5-carbohydrazide*, **3d** Yellow solid. Yield: 80%; m.p. 229–230 °C. ^1^H NMR (600 MHz, DMSO-d_6_) δ 12.25 (bs, NH), 9.75 (bs, 1H, OH), 8.08 (s, 1H, CH=N), 7.38 (d, *J* = 1.9 Hz, 1H, H-2), 7.22 (dd, *J* = 1.9, 8.2 Hz, 1H, H-6), 6.90 (d, *J* = 8.2 Hz, 1H, H-5), 3.89 (s, 3H, OMe), 2.97 (s, 3H, CH3). ^13^C NMR (151 MHz, DMSO-d_6_) δ 163.19 (C=O), 159.64 (C-5), 149.44 (C-3-Ar), 148.08 (C-4-Ar), 146.16 (CH=N), 135.40 (C-4), 124.69 (C-1-Ar), 122.32 (C-6-Ar), 115.80 (C-5-Ar), 110.15 (C-2-Ar), 55.51 (OCH_3_), 15.08 (CH_3_). HRMS (ESI) *m*/*z*: calcd: [M+H]^+^ 393.070287. Found: [M+H]^+^ 393.0694.

#### 3.1.2. General Procedure for the Synthesis of **5a**–**k**

A stirred solution of appropriate carbaldehydes **2a**–**k** (2.0 mmol) in abs. ethanol was added to a solution of benzenesulfonohydrazide **4** (2.0 mmol) in absolute ethanol. For the preparation of the compound **5k**, we used cinnamaldehyde **2k**. The solution was refluxed for 1–3 h. Treating the reaction mixture with a catalytic amount of *p*-toluenesulfonic acid (PTSA; 10 mol%) in refluxing ethanol produced the corresponding sulfonyl hydrazones in a suitable yield after half the reaction time. The solid product formed was collected by filtration and recrystallized with ethanol.

*N’-[(E)-(4-chloro-2-oxo-2H-1-benzopyran-3-yl)-methylidene]benzenesulfonohydrazide,***5a** Yellow solid. Yield: 75%; m.p. 163–165 °C. ^1^H NMR (600 MHz, DMSO-d_6_) δ 12.04 (s, 1H, NH), 7.98 (s, 1H, CH=N), 7.95 (dd, *J* = 1.5, 8.3 Hz, 1H, H-5), 7.91 (dd, *J* = 1.3, 8.4 Hz, 2H, H-o), 7.72 (ddd, *J* = 1.4, 7.4, 8.3 Hz, 1H, H-7), 7.69 (tt, *J* = 1.6, 7.4 Hz, 1H, H-p), 7.64 (tt, *J* = 1.6, 7.7 Hz, 2H, H-m), 7.47 (ddd, *J* = 1.2, 6.6, 7.8 Hz, 1H, H-6), 7.46 (d, *J* = 8.3 Hz, 1H, H-8). ^13^C NMR (151 MHz, DMSO-d_6_) δ 157.81 (C=O), 151.28 (C-8a), 144.57 (C-4), 140.18 (CH=N), 138.87 (C-i), 133.72 (C-7), 133.32 (C-p), 129.30 (C-m), 127.38 (C-o), 126.14 (C-5), 125.35 (C-6), 118.70 (C-3), 118.29 (C-4a), 116.58 (C-8). HRMS (ESI) *m/z*: calcd: [M+H]^+^ 363.020081. Found: [M+H]^+^ 363.02012.
*N’-[(E)-(5-methoxy-1-methyl-1H-indol-3-yl)methylidene]benzenesulfonohydrazide,***5b** Yellow solid. Yield: 83%; m.p. 190–191 °C. ^1^H NMR (600 MHz, DMSO-d_6_) δ 10.93 (s, 1H, NH), 8.05 (s, 1H, CH=N), 7.92 (dd, *J* = 1.5, 7.0 Hz, 2H, H-o), 7.64 (s, 1H, H-2), 7.63 (tt, *J* = 1.4, 7.4 Hz, 1H, H-p), 7.59 (tt, *J* = 1.7, 7.3 Hz, 2H, H-m), 7.44 (d, *J* = 2.5 Hz, 1H, H-4), 7.35 (d, *J* = 8.9 Hz, 1H, H-7), 6.85 (dd, *J* = 2.6, 8.9 Hz, 1H, H-6), 3.75 (s, 3H, OCH_3_), 3.73 (s, 3H, NCH_3_). ^13^C NMR (151 MHz, DMSO-d_6_) δ 154.68 (C-5), 145.35 (CH=N), 139.16 (C-i), 134.39 (C-2), 132.86 (C-p), 132.52 (C-7a), 129.09 (C-m), 127.34 (C-o), 124.87 (C-3a), 112.58 (C-6), 111.09 (C-7), 109.62 (C-3), 103.21 (C-4), 55.21 (OCH_3_), 32.94 (NCH_3_). HRMS (ESI) *m/z*: calcd: [M+H]^+^ 344.106338. Found: [M+H]^+^ 344.10625.
*N’-((E)-[5-(benzyloxy)-1H-indol-3-yl]methylidenebenzenesulfonohydrazide,***5c** Yellow solid. Yield: 87%; m.p. 208–209 °C. ^1^H NMR (600 MHz, DMSO-d_6_) δ 11.41 (s, 1H, NH-indol), 10.95 (s, 1H, NH), 8.08 (s, 1H, CH=N), 7.92 (d, *J* = 7.4 Hz, 2H, H-o), 7.68 (s, 1H, H-2), 7.61 (t, *J* = 6.8 Hz, 1H, H-p), 7.55–7.56 (m, 3H, H-m and H-4), 7.52 (d, *J* = 7.3 Hz, 2H, H-o), 7.42 (t, *J* = 6.9 Hz, 2H, H-m), 7.35 (t, *J* = 6.9 Hz, 1H, H-p), 7.29 (d, *J* = 8.5 Hz, 1H, H-7), 6.87 (d, *J* = 8.2 Hz, 1H, H-6), 5.03 (s, 2H, CH_2_). ^13^C NMR (151 MHz, DMSO-d_6_) δ 153.40 (C-5), 145.82 (CH=N), 139.22 (C-i), 137.38 (C-i), 132.83 (C-p), 132.01 (C-7a), 131.10 (C-2), 129.04 (C-m), 128.51 (C-m), 127.90 (C-o), 127.85 (C-p), 127.33 (C-o), 124.48 (C-3a), 113.07 (C-6), 112.56 (C-7), 110.77 (C-3), 104.81 (C-4), 69.66 (CH_2_). HRMS (ESI) *m/z*: calcd: [M+H]^+^ 406.121988. Found: [M+H]^+^ 406.12167.
*N’-[(E)-(5-chloro-1H-indol-3-yl)methylidene]benzenesulfonohydrazide,***5d** Yellow solid. Yield: 81%; m.p. 183–184 °C. ^1^H NMR (600 MHz, DMSO-d_6_) δ 11.70 (bs, 1H, NH-indol), 11.05 (s, 1H, NH), 8.08 (s, 1H, CH=N), 7.92 (d, *J* = 7.0 Hz, 2H, H-o), 7.89 (d, *J* = 2.0 Hz, 1H, H-4), 7.80 (d, *J* = 2.7 Hz, 1H, H-2), 7.66 (t, *J* = 7.2 Hz, 1H, H-p), 7.62 (t, *J* = 7.3 Hz, 2H, H-m), 7.41 (d, *J* = 8.6 Hz, 1H, H-7), 7.17 (dd, *J* = 2.1, 8.6 Hz, 1H, H-6). ^13^C NMR (151 MHz, DMSO-d_6_) δ 144.88 (CH=N), 138.98 (C-i), 135.36 (C-7a), 133.02 (C-p), 131.94 (C-2), 129.14 (C-m), 127.37 (C-o), 125.09 (C-3a), 125.02 (C-5), 122.56 (C-6), 120.73 (C-4), 113.47 (C-7), 110.68 (C-3). HRMS (ESI) *m/z*: calcd: [M+H]^+^ 334.041151. Found: [M+H]^+^ 334.04123.
*N’-[(E)-(5-methoxy-1H-indol-3-yl)methylidene]benzenesulfonohydrazide,***5e** Yellow solid. Yield: 90%; m.p. 174–175 °C. ^1^H NMR (600 MHz, DMSO-d_6_) δ 11.40 (d, *J* = 2.0 Hz, 1H, NH-indol), 10.94 (s, 1H, NH), 8.08 (s, 1H, CH=N), 7.93 (td, *J* = 1.6, 6.5 Hz, 2H, H-o), 7.67 (d, *J* = 2.8 Hz, 1H, H-2), 7.64 (tt, *J* = 1.8, 7.3 Hz, 1H, H-p), 7.60 (tt, *J* = 1.7, 7.1 Hz, 2H, H-m), 7.43 (d, *J* = 2.5 Hz, 1H, H-4), 7.28 (d, *J* = 8.8 Hz, 1H, H-7), 6.79 (dd, *J* = 2.6, 8.8 Hz, 1H, H-6), 3.74 (s, 3H, CH_3_). ^13^C NMR (151 MHz, DMSO-d_6_) δ 154.38 (C-5), 145.82 (CH), 139.17 (C-i), 132.85 (C-p), 131.79 (C-7a), 130.95 (C-2), 129.08 (C-m), 127.35 (C-o), 124.46 (C-3a), 112.65 (C-6), 112.55 (C-7), 110.77 (C-3), 103.03 (C-4), 55.15 (CH_3_). HRMS (ESI) *m/z*: calcd: [M+H]^+^ 330.090688. Found: [M+H]^+^ 330.09057.
*N’-[(E)-(2-nitrophenyl)methylidene]benzenesulfonohydrazide,***5f** Yellow solid. Yield: 79%; m.p. 148–149 °C. ^1^H NMR (600 MHz, DMSO-d_6_) δ 12.00 (s, 1H, NH), 8.29 (s, 1H, CH=N), 8.02 (dd, *J* = 1.2, 8.2 Hz, 1H, H-3), 7.89 (td, *J* = 1.5, 6.6 Hz, 2H, H-o), 7.81 (dd, *J* = 1.5, 7.9 Hz, 1H, H-6), 7.75 (dt, *J* = 1.0, 7.6 Hz, 1H, H-5), 7.69 (tt, *J* = 1.6, 7.3 Hz, 1H, H-p), 7.62–7.56 (m, 3H, H-m and H-4). ^13^C NMR (151 MHz, DMSO-d_6_) δ 147.88 (CH=N), 142.71 (C-2), 138.92 (C-i), 133.82 (C-5), 133.30 (C-p), 130.79 (C-4), 129.43 (C-m), 128.00 (C-1), 127.90 (C-6), 127.14 (C-o), 124.71 (C-3). HRMS (ESI) *m/z*: calcd: [M+H]^+^ 306.054302. Found: [M+H]^+^ 306.0535.
*N’-[(E)-(4-nitrophenyl)methylidene]benzenesulfonohydrazide,***5g** Yellow solid. Yield: 92%; m.p. 166–167 °C. ^1^H NMR (600 MHz, DMSO-d_6_) δ 12.01 (s, 1H, NH), 8.23 (td, *J* = 2.1, 9.3 Hz, 2H, H-3 and H-5), 8.03 (s, 1H, CH=N), 7.90 (td, *J* = 1.5, 6.7 Hz, 2H, H-o), 7.83 (td, *J* = 1.4, 8.9 Hz, 2H, H-2 and H-6), 7.68 (tt, *J* = 1.6, 7.4 Hz, 1H, H-p), 7.62 (tt, *J* = 1.5, 7.5 Hz, 2H, H-m). ^13^C NMR (151 MHz, DMSO-d_6_) δ 147.90 (C-p), 144.62 (CH=N), 139.78 (C-i), 138.85 (C-i), 133.32 (C-p), 129.42 (C-m), 127.76 (C-2 and C-6), 127.15 (C-o), 124.09 (C-3 and C-05). HRMS (ESI) *m/z*: calcd: [M+H]^+^ 306.054302. Found: [M+H]^+^ 306.0535.
*N’-[(E)-(3-hydroxy-4-methoxyphenyl)methylidene]benzenesulfonohydrazide,***5h** Yellow solid. Yield: 75%; m.p. 129–13 °C. ^1^H NMR (600 MHz, DMSO-d_6_) δ 11.29 (s, 1H, NH), 9.25 (bs, 1H, OH), 7.86 (td, *J* = 1.6, 6.6 Hz, 2H, H-o), 7.77 (s, 1H, CH=N), 7.66 (tt, *J* = 1.7, 7.4 Hz, 1H, H-p), 7.60 (tt, *J* = 1.6, 11.6 Hz, 2H, H-m), 7.05 (d, *J* = 1.3 Hz, 1H, H-2), 6.89–6.92 (m, 2H, H-5 and H-6), 3.76 (s, 3H, OCH_3_). ^13^C NMR (151 MHz, DMSO-d_6_) δ 149.80 (C-4), 147.58 (CH=N), 146.76 (C-34), 139.13 (C-i), 133.01 (C-p), 129.24 (C-m), 127.16 (C-o), 126.46 (C-1), 120.14 (C-6), 111.87 (C-2), 111.75 (C-5), 55.56 (OCH_3_). HRMS (ESI) *m/z*: calcd: [M+H]^+^ 307.074703. Found: [M+H]^+^ 307.0738.
*N’-[(E)-(4-chlorophenyl)methylidene]benzenesulfonohydrazide,***5i** White solid. Yield: 80%; m.p. 161–163 °C. ^1^H NMR (600 MHz, DMSO-d_6_) δ 11.64 (s, 1H, NH), 7.91 (s, 1H, CH=N), 7.88 (td, *J* = 1.5, 6.6 Hz, 2H, H-o), 7.67 (tt, *J* = 1.6, 7.1 Hz, 1H, H-p), 7.61 (tt, *J* = 1.5, 7.2 Hz, 2H, H-m), 7.58 (td, *J* = 2.2, 9.1 Hz, 2H, H-2 and H-6), 7.45 (td, *J* = 2.2, 9.1 Hz, 2H, H-3 and H-5). ^13^C NMR (151 MHz, DMSO-d_6_) δ 145.90 (CH=N), 138.95 (C-i), 134.60 (C-4), 133.16 (C-p), 132.56 (C-1), 129.32 (C-m), 128.93 (C-3 and C-5), 128.44 (C-2 and C-6), 127.18 (C-o). HRMS (ESI) *m/z*: calcd: [M+H]^+^ 295.030252. Found: [M+H]^+^ 295.03044.
*N’-[(E)-(3,4-dimethoxyphenyl)methylidene]benzenesulfonohydrazide,***5j** White solid. Yield: 87%; m.p. 150–152 °C. ^1^H NMR (600 MHz, DMSO-d_6_) δ 11.33 (s, 1H, NH), 7.88 (td, *J* = 2.1, 7.7 Hz, 2H, H-o), 7.83 (s, 1H, CH=N), 7.66 (tt, *J* = 1.7, 11.1 Hz, 1H, H-p), 7.61 (tt, *J* = 1.6, 7.5 Hz, 2H, H-m), 7.12 (d, *J* = 1.9 Hz, 1H, H-2), 7.08 (dd, *J* = 1.9, 8.3 Hz, 1H, H-6), 6.95 (d, *J* = 8.4 Hz, 1H, H-5), 3.76 (s, 3H, OCH_3_), 3.76 (s, 3H, OCH_3_). ^13^C NMR (151 MHz, DMSO-d_6_) δ 150.66 (C-4), 148.90 (C-3), 147.45 (CH=N), 139.01 (C-i), 133.05 (C-p), 129.21 (C-m), 127.26 (C-o), 126.36 (s, 1C), 121.00 (C-6), 111.49 (C-5), 108.58 (C-2), 55.56 (OCH_3_), 55.42 (OCH_3_). HRMS (ESI) *m/z*: calcd: [M+H]^+^ 321.090353. Found: [M+H]^+^ 321.0895.
*N’-[(1E,2E)-3-phenylprop-2-en-1-ylidene]benzenesulfonohydrazid,***5k** White solid. Yield: 85%; m.p. 168–170 °C. ^1^H NMR (600 MHz, DMSO-d_6_) δ 11.50 (s, 1H, NH), 7.83–7.85 (m, 2H, H-o), 7.73 (d, *J* = 9.2 Hz, 1H, H-1), 7.66–7.68 (m, 1H, H-p), 7.60–7.63 (m, 2H, H-m), 7.54–7.56 (m, 2H, H-o), 7.33–7.36 (m, 2H, H-m), 7.28–7.31 (m, 1H, H-p), 6.95 (d, *J* = 16.1 Hz, 1H, H-3), 6.84 (dd, *J* = 9.2, 16.1 Hz, 1H, H-2). ^13^C NMR (151 MHz, DMSO-d_6_) δ 149.48 (C-1), 139.38 (C-3), 139.14 (C-i), 135.65 (C-i), 133.06 (C-p), 129.32 (C-m), 128.94 (C-p), 128.80 (C-m), 127.15 (C-o), 127.12 (C-o), 124.68 (C-2). HRMS (ESI) *m/z*: calcd: [M+H]^+^ 287.084874. Found: [M+H]^+^ 287.0842.

### 3.2. In Vitro Antimycobacterial Activity

The in vitro antimycobacterial activity of the tested compounds was assessed according to the EUCAST broth microdilution reference method for MIC determination [78]. The *M. tuberculosis* H37Rv strain (ATCC 27294) was cultured at 37 °C in Loewenstein-Jensen medium until log phase growth; then, a cell suspension was prepared at a concentration of approximately 2 × 10^6^ cells/mL and further diluted 1:20 in Middlebrook 7H9 medium with 10% OADC (oleic acid-albumin-dextrose-catalase) (Becton Dickinson and Co., Sparks, MD, USA). Ninety-six-well microplates were used. The Middlebrook 7H9 medium was added dropwise at the appropriate concentration of the test compound (range of 0.25 to 32 mg/L) and *M. tuberculosis* suspension. Ethambutol and isoniazid were used as controls. Reading was performed after 7, 14, and 21 days of incubation at 37 °C using an inverted mirror. The MIC was the lowest concentration without visual growth.

### 3.3. In Vitro Cytotoxicity Screening

The cytostatic activity of the investigational compounds was determined using a standard MTT-based colorimetric assay for evaluating cell viability [79,80]. HEK-293 and CCL-1 cells were harvested and seeded (100 µL/well) in 96-well plates at a density of 3 × 10^5^. Following a 24 h incubation, the cells were treated with serial dilutions of the tested compounds in the concentration range of 200.0–12.5 µM. Following an exposure time of 72 h, a filter-sterilized MTT substrate solution (5 mg/mL in PBS) was added to each well of the culture plate. A further 1–4 h incubation allowed the formation of purple insoluble precipitates of the formazan dye. The latter was dissolved in an isopropyl alcohol solution containing 5% formic acid for absorbance measurement at 550 nm. The collected absorbance values were blanked against MTT-and isopropanol solution and normalized to the mean value of the untreated control (100% cell viability).

### 3.4. Statistical Methods

Semi-logarithmic “dose-response” curves were computed using nonlinear regression in GraphPad Prism^®^ 8.0. The antiproliferative potential of the studied compounds was rated according to the calculated half-maximal inhibitory concentrations (IC_50_ values).

### 3.5. ADME/Tox Screening

ADME screenings were performed using the online tool SwissADME of the Swiss Institute of Bioinformatics (https://www.sib.swiss, accessed on 20 April 2022). The tool is based on multiple linear regression, binary classification, and support vector machine algorithms performed over large data sets of known inhibitors/non-inhibitors, as well as on substrates/non-substrates [81,82]. The web service ProTox-II was used to predict the toxicity of the synthesized compounds. The tool incorporates computer models based on chemical similarities and machine-learning algorithms [83,84]. The models were pre-trained on specific databases of real data to estimate the balanced accuracy and the specific confidence rate at each classification.

### 3.6. Molecular Docking

Molecular docking studies were performed using Molecular Operating Environment developed by the Chemical Computing Group, version 2016.08 (MOE, https://www.chemcomp.com/MOE-Molecular_Operating_Environment.htm, accessed on 20 April 2022). Before proceeding with docking, all the ligands and water molecules were removed from the crystal structures, except the co-factor NAD^+^. To position the missing hydrogen atoms, the “Protonate 3D” tool of MOE was tutor to have assigned the correct ionization states assigned to the protein structure. The docking procedure was implemented via the “Docking” module in MOE. No changes were made in the default settings of the docking procedure. Docking was performed within a rigid receptor, and the top 30 poses ranked by London dG were kept. For further analysis of the molecular docking results, the “Ligand Interactions” MOE tool was used to visualize the protein–ligand interactions in the active site of the complexes.

## 4. Conclusions

In this study, we present the synthesis and investigation of the substituted sulfonyl hydrazones scaffolds **5a**–**k** and 4-methyl-1,2,3-thiadiazole-containing hydrazone derivatives **3a**–**d**, looking for the most effective compounds as *M. tuberculosis* growth inhibitors with low cytotoxicity and a highly selective index, that would reduce or eliminate adverse effects. The new compound **3d** displayed antimycobacterial activity at a submicromolar concentration level with the lowest MIC of 0.0730 μM against *M. tuberculosis* H37Rv and remarkably minimal associated cytotoxicity in the normal human embryonic kidney cell line HEK-293T and mouse fibroblast cell line CCL-1. It was also found that the vanillin, cinnamyl, and *p*-nitrophenyl fragments in **3d**, **5h**, **5g**, and **5k**, as well as 4-methyl-1,2,3-thiadiazole scaffolding in **3a**–**d** and **5h**, may be pharmacophores with antimycobacterial activity much higher than other compounds tested. The in silico ADME study revealed that all compounds had suitable bioavailability and fraction absorption at high levels of gastrointestinal absorption. All the compounds that form the collection seem to be suitable drug-like molecules in terms of their satisfactory membrane permeability and oral bioavailability. Their predicted toxicity properties are a prerequisite for considering the new compounds as effective and safe and with potential for TB treatment. The results of the molecular docking studies agree with experimental studies focused on the significance of the synthesized compounds as potential growth inhibitors of *M. tuberculosis*. All most active compounds displayed interactions with critical residues. Thus, we consider that the 4-methyl-1,2,3-thiadiazole derivatives and sulfonyl hydrazones are promising scaffolds for antitubercular drug discovery that prompt further studies on their mechanism of action to completely validate InhA as the main molecular target.

## Figures and Tables

**Table 1:** Antimycobacterial activity of hydrazones with a 4-methyl-1,2,3-thiadiazole scaffold (**3a**–**d**) and *N*-substituted sulfonyl hydrazones (**5a**–**k**) and cytotoxicity of the tested substances in HEK-293 (human embryonic kidney 293) cells and CCL-1 mouse fibroblast cell line.

Compd.	Formula	MIC ^a^ (µM)	IC_50_ (µM) ^b^ CCL-1	IC_50_ (µM) ^c^ HEK-293	SI ^d^ CCL-1	SI ^d^ HEK-293
**3a**	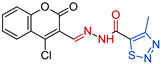	0.3914	32.9 ± 6.2	51.2 ± 4.7	84	130
**3b**	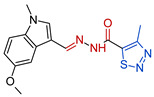	0.3294	818.3 ± 23.7	361 ± 11.3	2487	1244
**3c**	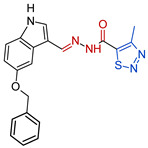	0.1744	390.8 ± 12.0	713.5 ± 18.5	2242	4093
**3d**	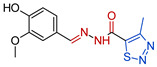	0.0730	256.7 ± 13.3	217.5 ± 17.2	3516	2979
**5a**	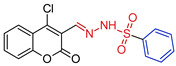	0.1814	48.0 ± 5.9	83.9 ± 4.0	267	463
**5b**	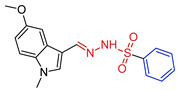	0.2027	2.9 ± 0.3	16.2 ± 2.7	14	82
**5c**	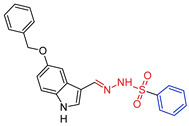	0.3434	321.4 ± 16.2	32.3 ± 1.5	945	95
**5d**	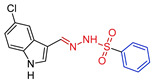	0.1669	13.8 ± 0.9	16.9 ± 3.4	11	97
**5e**	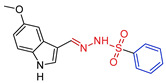	0.1647	5.1 ± 1.1	15.6 ± 4.1	31	95
**5f**	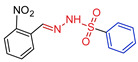	0.3053	72.4 ± 5.8	100.1 ± 2.1	237	327
**5g**	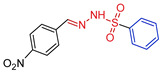	0.0763	191 ± 13.2	138.3 ± 7.4	1812	1819
**5h**	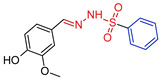	0.3203	223 ± 11.5	150.2 ± 5.1	469	696
**5i**	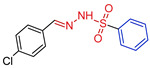	0.1473	131.4 ± 11.4	100.1 ± 9.3	892	679
**5j**	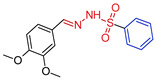	0.3210	83.5 ± 7.0	47.4 ± 6.8	146	260
**5k**	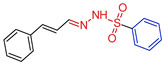	0.0716	240 ± 9.6	158.5 ± 11.2	3380	2216
**INH**		0.0343	-	-	-	-

^a^ Antimycobacterial activity against *M. tuberculosis* strain H37Rv; minimum inhibitory concentration-MIC (µM) was defined as the lowest concentration resulting in a complete; ^b^ in vitro cytotoxicity against human embryonal kidney cell line HEK-293T, IC_50_ (µM). ^c^ In vitro cytotoxicity against the mouse fibroblast cell line—CCL-1, IC_50_ (µM). ^d^ Selectivity index—SI ratio = IC_50_/MIC by the MTT assay. INH, isoniazid.

**Table 2:** Chemical properties of the compounds.

Scheme	MW ^1^ (g/mol)	TPSA ^2^ (Å^2^)	HBA ^3^	HBD ^4^	Rotatable Bonds	Moriguchi’s LogP	Water Solubility
**3a**	348.76430	125.69	6	1	4	1.37	Poorly soluble
**3b**	329.37890	109.64	5	1	5	0.67	Moderately soluble
**3c**	391.44628	120.50	5	2	7	1.64	Moderately soluble
**3d**	292.31368	124.94	6	2	5	0.03	Soluble
**5a**	362.78754	97.12	5	1	4	2.15	Poorly soluble
**5b**	343.40018	81.07	4	1	5	1.44	Moderately soluble
**5c**	405.46950	91.93	4	2	7	2.37	Poorly soluble
**5d**	333.79268	82.70	3	2	4	2.01	Poorly soluble
**5e**	329.37360	91.93	4	2	5	1.20	Moderately soluble
**5f**	305.30914	112.73	5	1	5	1.86	Moderately soluble
**5g**	305.30914	112.73	5	1	5	1.86	Moderately soluble
**5h**	306.33696	96.37	5	2	5	1.23	Moderately soluble
**5i**	294.75664	66.91	3	1	4	2.61	Moderately soluble
**5j**	320.36354	85.37	5	1	6	1.49	Moderately soluble
**5k**	286.34886	66.91	3	1	5	2.52	Moderately soluble
**INH**	137.14	68.01	3	2	2	−0.47	Soluble
**EMB**	204.31	64.52	4	4	9	0.18	Soluble

^1^ Molecular weight; ^2^ topological polar surface area (TPSA); ^3^ hydrogen bond acceptors; ^4^ hydrogen bond donors. INH, isoniazid; EMB, ethambutol.

**Table 3:** Pharmacokinetics and drug-likeness prediction.

			Pharmacokinetics							Drug Likeness			
Compound	GI Absorbtion	BBB Perm.	P-gp Substrate	CYP1A2 Inhib.	CYP2C19 Inhib.	CYP2C9 Inhib.	CYP2D6 Inhib.	CYP3A4 Inhib.	Log *K*p (cm/s)	Lipinski	Ghose	Veber	Bio. Score
**3a**	high	no	no	yes	yes	no	no	no	−6.56	yes	yes	yes	0.55
**3b**	high	no	no	yes	yes	yes	no	yes	−6.80	yes	yes	yes	0.55
**3c**	high	no	no	yes	yes	yes	no	yes	−6.08	yes	yes	yes	0.55
**3d**	high	no	no	no	no	no	no	no	−6.88	yes	yes	yes	0.55
**5a**	high	no	no	no	yes	no	no	no	−6.34	yes	yes	yes	0.55
**5b**	high	no	no	no	yes	yes	no	yes	−6.57	yes	yes	yes	0.55
**5c**	high	no	no	yes	yes	yes	yes	yes	−5.86	yes	yes	yes	0.55
**5d**	high	no	no	yes	yes	yes	no	yes	−6.01	yes	yes	yes	0.55
**5e**	high	no	no	yes	yes	yes	no	no	−6.45	yes	yes	yes	0.55
**5f**	high	no	yes	yes	no	no	no	no	−6.49	yes	yes	yes	0.55
**5g**	high	no	yes	yes	no	no	no	no	−6.49	yes	yes	yes	0.55
**5h**	high	no	no	no	no	no	no	no	−6.67	yes	yes	yes	0.55
**5i**	high	yes	no	yes	yes	yes	no	no	−5.86	yes	yes	yes	0.55
**5j**	high	no	no	yes	yes	yes	no	no	−6.51	yes	yes	yes	0.55
**5k**	high	yes	no	yes	yes	yes	no	no	−5.95	yes	yes	yes	0.55
**INH**	high	no	no	no	no	no	no	no	−7.63	yes	no:3 viol.	yes	0.55
**EMB**	high	no	no	no	no	no	no	no	−7.60	yes	yes	yes	0.55

INH, isoniazid; EMB, ethambutol.

**Table 4:** In silico toxicity prediction.

No.	Compound	Oral Toxicity Class	Predicted LD50 (mg/kg)	Organ Toxicity (Hepatotoxicity)		Carcinogenicity		Immunotoxicity		Mutagenicity		Cytotoxicity	
				Pr	Prob	Pr	Prob	Pr	Prob	Pr	Prob	Pr	Prob
**1**	**3a**	III	187	A	0.61	I	0.55	I	0.97	A	0.52	I	0.69
**2**	**3b**	IV	1120	A	0.51	I	0.56	A	0.78	A	0.54	I	0.67
**3**	**3c**	V	4920	A	0.63	I	0.52	I	0.82	A	0.55	I	0.65
**4**	**3d**	V	4920	A	0.60	A	0.56	I	0.55	A	0.56	I	0.64
**5**	**5a**	IV	500	I	0.53	I	0.60	I	0.99	I	0.68	I	0.71
**7**	**5b**	IV	500	I	0.50	I	0.51	I	0.92	I	0.50	I	0.72
**8**	**5c**	IV	500	I	0.50	I	0.53	I	0.99	I	0.55	I	0.78
**9**	**5d**	IV	500	I	0.55	I	0.58	I	0.99	I	0.68	I	0.77
**10**	**5e**	IV	500	I	0.50	I	0.50	I	0.94	I	0.53	I	0.81
**11**	**5f**	IV	500	I	0.50	A	0.51	I	0.99	I	0.55	I	0.79
**12**	**5g**	IV	500	A	0.52	A	0.57	I	0.99	I	0.63	I	0.73
**13**	**5h**	IV	500	I	0.53	A	0.55	I	0.95	I	0.59	I	0.87
**14**	**5i**	IV	500	I	0.63	I	0.60	I	0.99	I	0.66	I	0.70
**15**	**5j**	IV	500	I	0.51	A	0.54	I	0.99	I	0.55	I	0.88
**16**	**5k**	III	283	I	0.59	A	0.51	I	0.99	I	0.59	I	0.69
	**INH**	III	133	A	0.94	A	0.98	I	0.99	I	0.63	I	0.81
	**EMB**	IV	998	A	0.63	I	0.56	I	0.99	I	0.95	I	0.72

Class I: death after swallowing (LD_50_ ≤ 5); Class II: death after swallowing (5 < LD_50_ ≤ 50); Class III: toxic after swallowing (50 < LD_50_ ≤ 300); Class IV: harmful after swallowing (300 < LD_50_ ≤ 2000); Class V: may be harmful after swallowing (2000 < LD_50_ ≤ 5000) and Class VI: non-toxic (LD_50_ > 5000) [72]; Pr—predicted: A—active; I—inactive.

**Table 5:** Docking results for compounds **3a**–**d** and **5a**–**k.**

Compound	2X22 E_Score1 * (kcal/mol)	4TZK E_Score1 * (kcal/mol)
**3a**	−10.93 (13)	−11.56 (14)
**3b**	−10.02 (15)	−11.10 (15)
**3c**	−11.09 (9)	−11.62 (13)
**3d**	−12.19 (2)	−12.38 (6)
**5a**	−11.06 (10)	−12.15 (9)
**5b**	−10.99 (12)	−12.30 (7)
**5c**	−10.73 (14)	−12.66 (4)
**5d**	−11.60 (5)	−12.19 (8)
**5e**	−12.03 (3)	−14.67 (1)
**5f**	−11.29 (7)	−11.88 (10)
**5g**	−11.98 (4)	−12.80 (3)
**5h**	−11.43 (6)	−12.49 (5)
**5i**	−11.18 (8)	−11.87 (11)
**5j**	−11.06 (11)	−11.75 (12)
**5k**	−12.36 (1)	−12.83 (2)
**INH**	−9.18 (16)	−8.51 (16)

* E_score1—the energy score from rescoring stage 1, in kcal/mol.

## Data Availability

All obtained data are presented in this article and supplementary files.

## Supplementary Materials

The following supporting information can be downloaded at: https://www.mdpi.com/article/10.3390/antibiotics11050562/s1. Supplementary Files: ^1^H NMR, ^13^C NMR, and HRMS spectra and raw data.
