# Supplementary material for: Development of New Antimycobacterial Sulfonyl Hydrazones and 4-Methyl-1,2,3-thiadiazole-Based Hydrazone Derivatives
Source: Antibiotics (Basel). 2022 Apr 22;11(5):562. doi: 10.3390/antibiotics11050562 (PMC9137698; doi:10.3390/antibiotics11050562)
Supplement: Supplementary file 1 [file antibiotics-11-00562-s001.zip › supporting R2 v2. pdf (1).pdf]

Supporting Information

Synthesis, antimycobacterial activity, cytotoxicity, in silico ADME and docking studies of sulfonyl hydrazones and 4-methyl-1,2,3-thiadiazole based hydrazone derivatives

Violina T. Angelova[1*], Tania Pencheva[2], Nikolay Vassilev[3], Elena K-Yovkova[4], Rositsa Mihaylova [5], Boris Petrov[1], Violeta Valcheva[6*]

[1]Department of Chemistry, Faculty of Pharmacy, Medical University-Sofia, Bulgaria;

[2]Department of QSAR and Molecular Modeling, Institute of Biophysics and Biomedical Engineering, Bulgarian Academy of Sciences, Sofia, Bulgaria

[3]Laboratory “Nuclear Magnetic Resonance“, Institute of Organic Chemistry with Centre of Phytochemistry, Bulgarian Academy of Sciences, Sofia, Bulgaria;

[4]Faculty of Computer Systems and Technilogies, Technical University, Sofia, Bulgaria;

[5]Laboratory “Drug metabolism and drug toxicity”, Department “Pharmacology, Pharmacotherapy and Toxicology”, Faculty of Pharmacy, Medical University, Sofia, Bulgaria

[6]Institute of Microbiology, Bulgarian Academy of Sciences, Sofia, Bulgaria.

^{1}H NMR, ^{13}C NMR and HRMS spectra

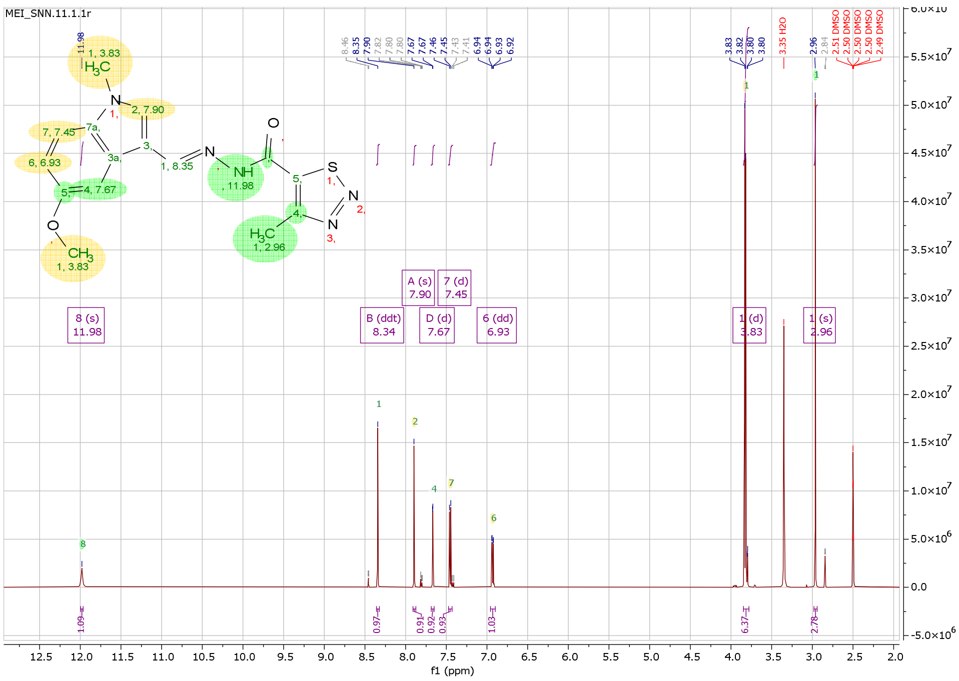

Figure S1. ^{1}**H NMR spectrum of** ***N'-[(E)-(5-methoxy-1-methyl-1H-indol-3-yl)methylidene]-4-methyl-1,2,3-thiadiazole-5-carbohydrazide, 3b in DMSO-d6***

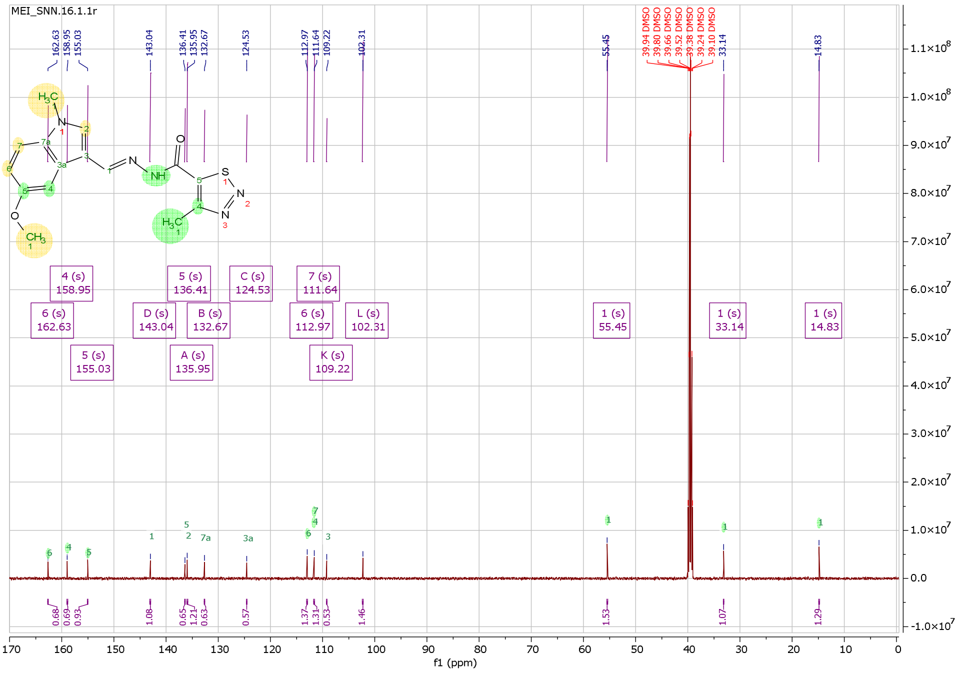

Figure S2. ^{13}**C NMR spectrum of** ***N'-[(E)-(5-methoxy-1-methyl-1H-indol-3-yl)methylidene]-4-methyl-1,2,3-thiadiazole-5-carbohydrazide, 3b in DMSO-d6***

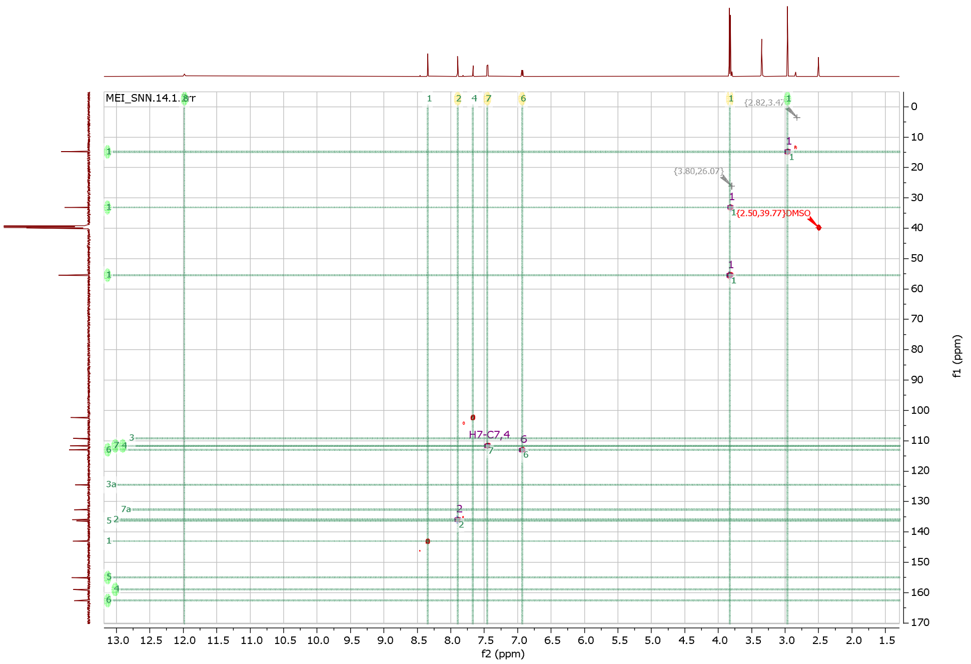

Figure S3. HSQC NMR spectrum of *N'-[(E)-(5-methoxy-1-methyl-1H-indol-3-yl)methylidene]-4-methyl-1,2,3-thiadiazole-5-carbohydrazide, 3b in DMSO-d6*

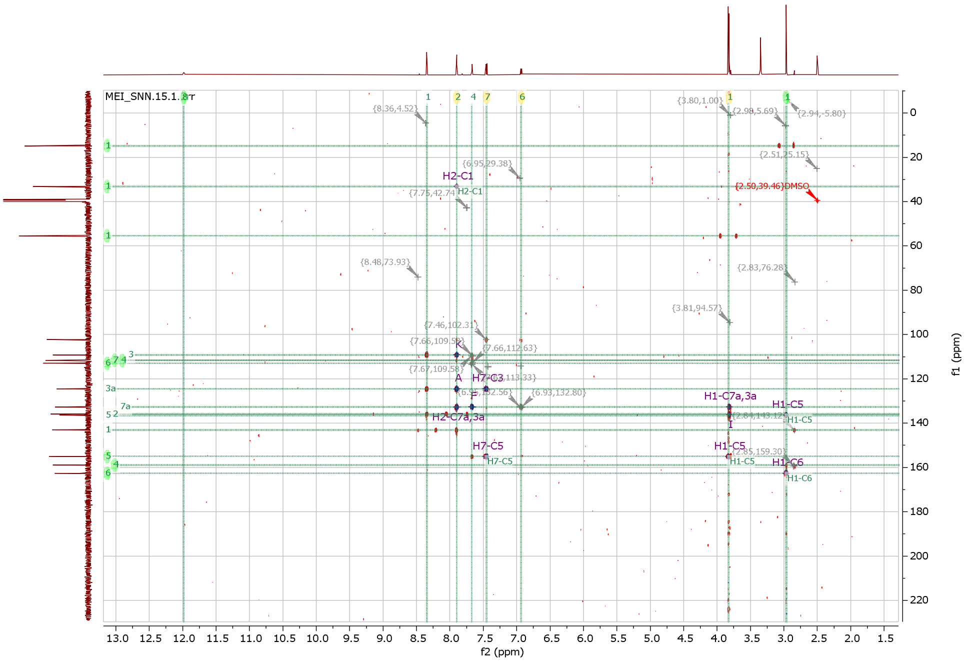

Figure S4. HMBC NMR spectrum of *N'-[(E)-(5-methoxy-1-methyl-1H-indol-3-yl)methylidene]-4-methyl-1,2,3-thiadiazole-5-carbohydrazide, 3b in DMSO-d6*

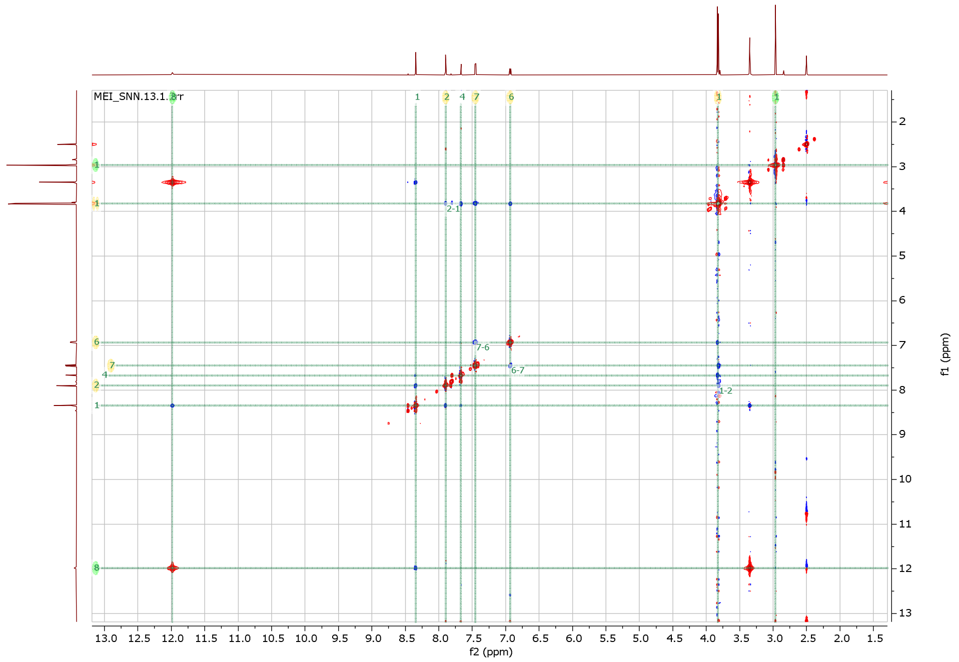

Figure S5. NOESY NMR spectrum of *N'-[(E)-(5-methoxy-1-methyl-1H-indol-3-yl)methylidene]-4-methyl-1,2,3-thiadiazole-5-carbohydrazide, 3b in DMSO-d6*

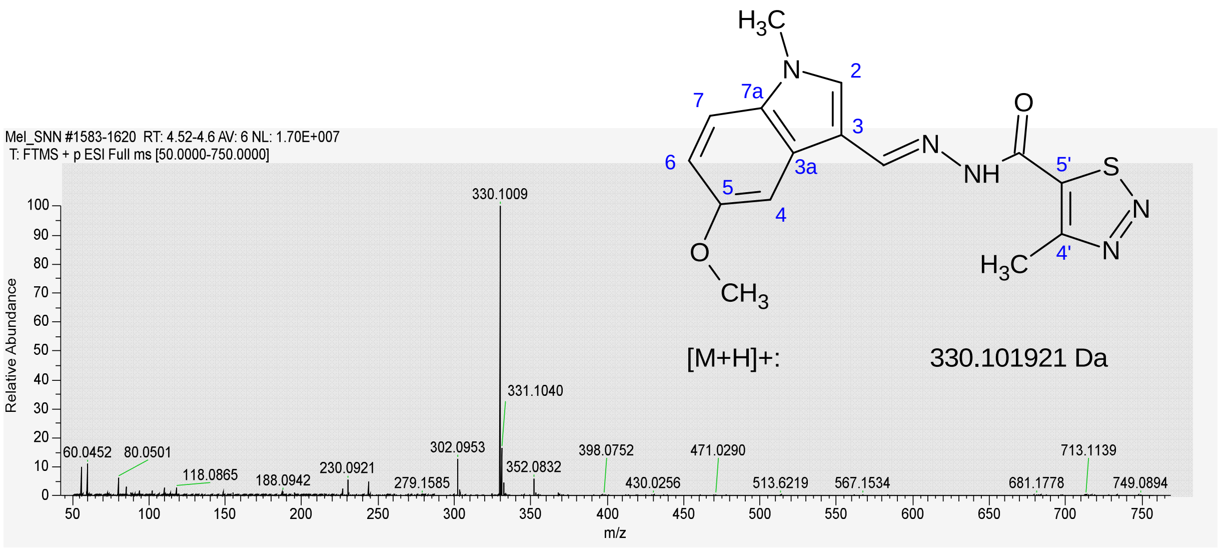

Figure S6. HRMS ***of N'-[(E)-(5-methoxy-1-methyl-1H-indol-3-yl)methylidene]-4-methyl-1,2,3-thiadiazole-5-carbohydrazide, 3b***

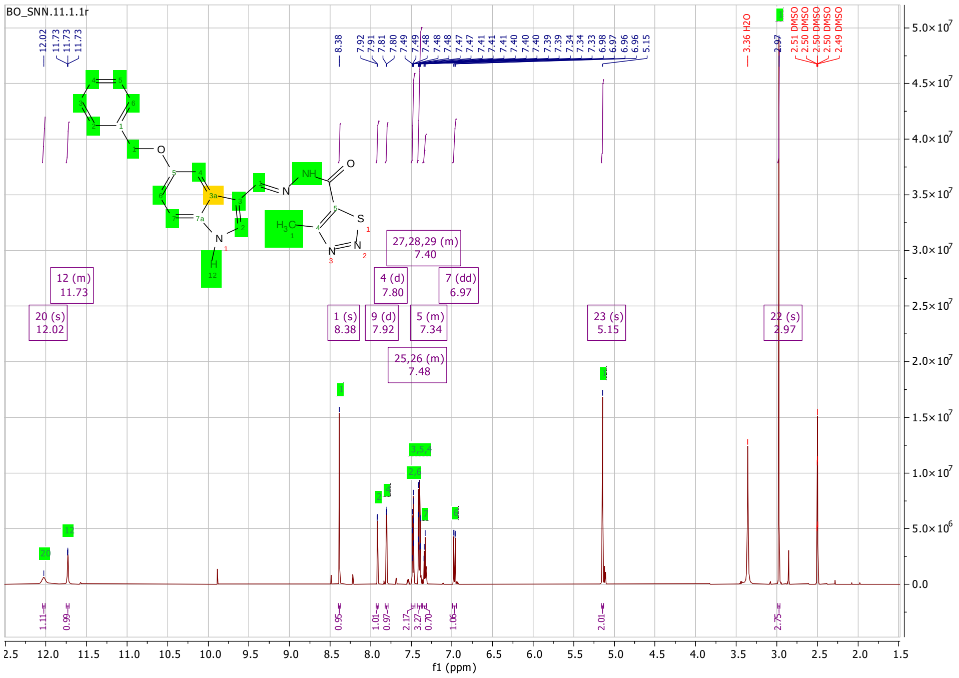

Figure S7. [1]**H NMR spectrum of** ***N'-{(E)-[5-(benzyloxy)-1H-indol-3-yl]methylidene}-4-methyl-1,2,3-thiadiazole-5-carbohydrazide, 3c in DMSO-d6***

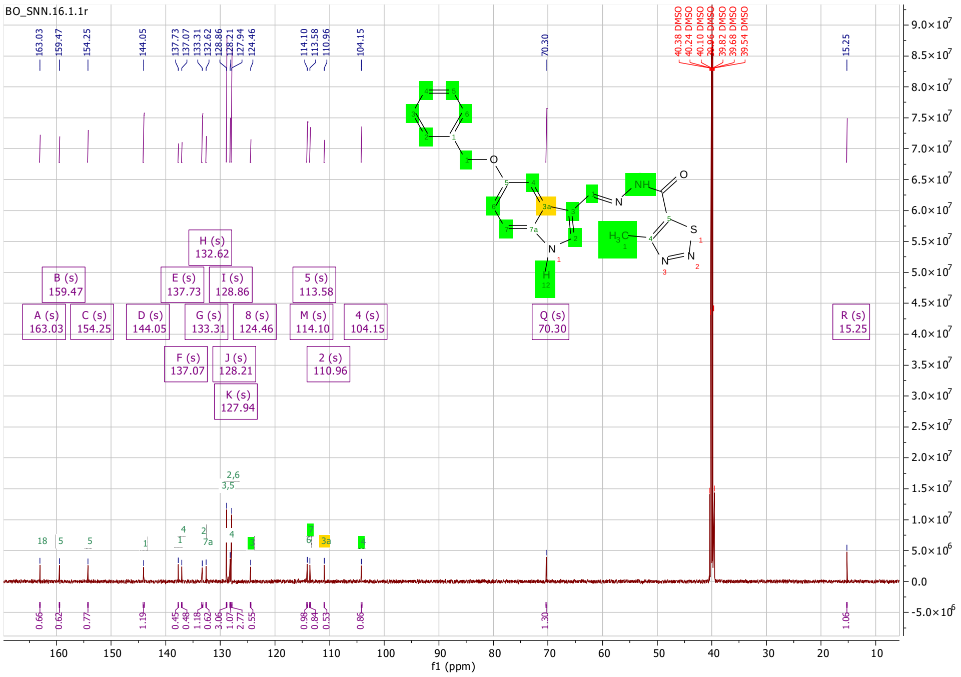

Figure S8. ^{13}**C NMR spectrum of** ***N'-{(E)-[5-(benzyloxy)-1H-indol-3-yl]methylidene}-4-methyl-1,2,3-thiadiazole-5-carbohydrazide, 3c in DMSO-d6***

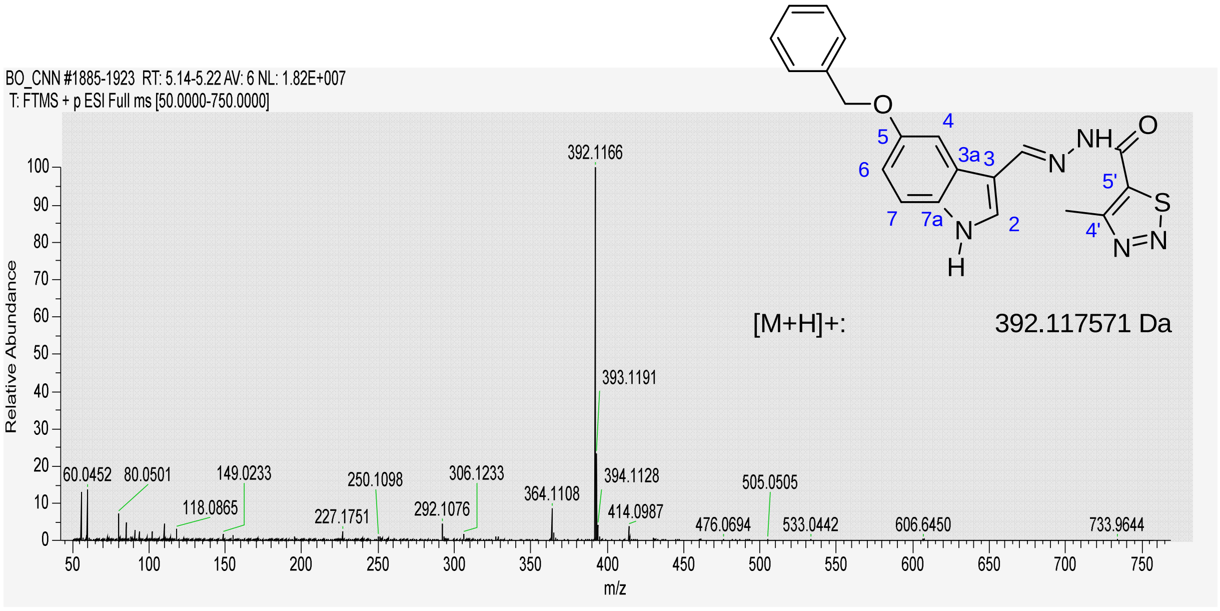

Figure S9. HRMS of ***N'-{(E)-[5-(benzyloxy)-1H-indol-3-yl]methylidene}-4-methyl-1,2,3-thiadiazole-5-carbohydrazide, 3c***

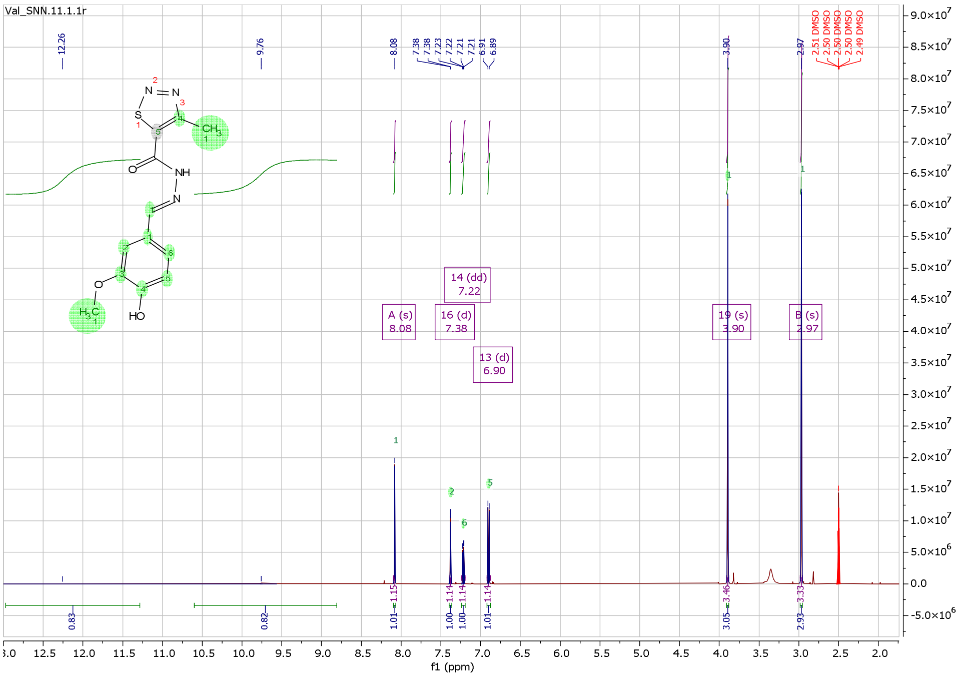

Figure S10. **^{1}H NMR spectrum of** ***N'-[(E)-(4-hydroxy-3-methoxyphenyl)methylidene]-4-methyl-1,2,3-thiadiazole-5-carbohydrazide, 3d in DMSO-d6***

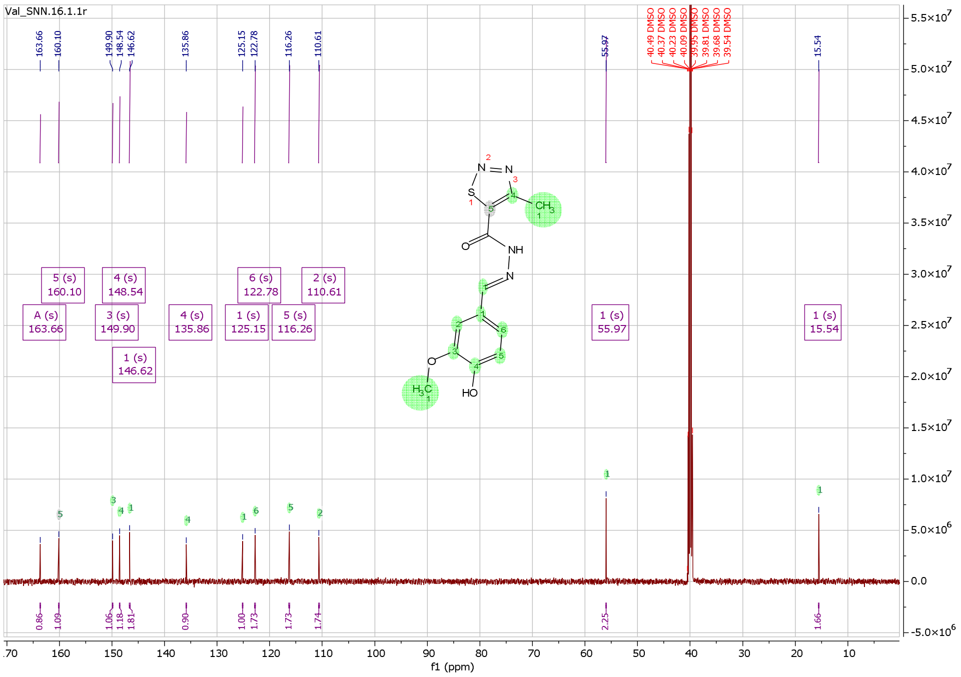

Figure S11. **^{13}C NMR spectrum of** ***N'-[(E)-(4-hydroxy-3-methoxyphenyl)methylidene]-4-methyl-1,2,3-thiadiazole-5-carbohydrazide, 3d in DMSO-d6***

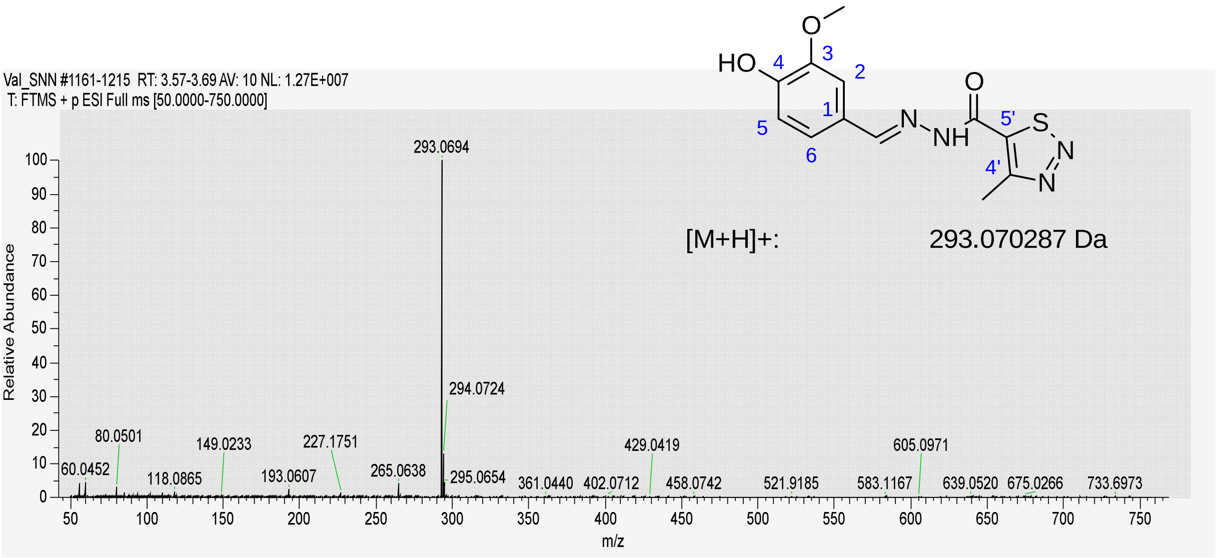

Figure S12. ***HRMS of N'-[(E)-(4-hydroxy-3-methoxyphenyl)methylidene]-4-methyl-1,2,3-thiadiazole-5-carbohydrazide, 3d***

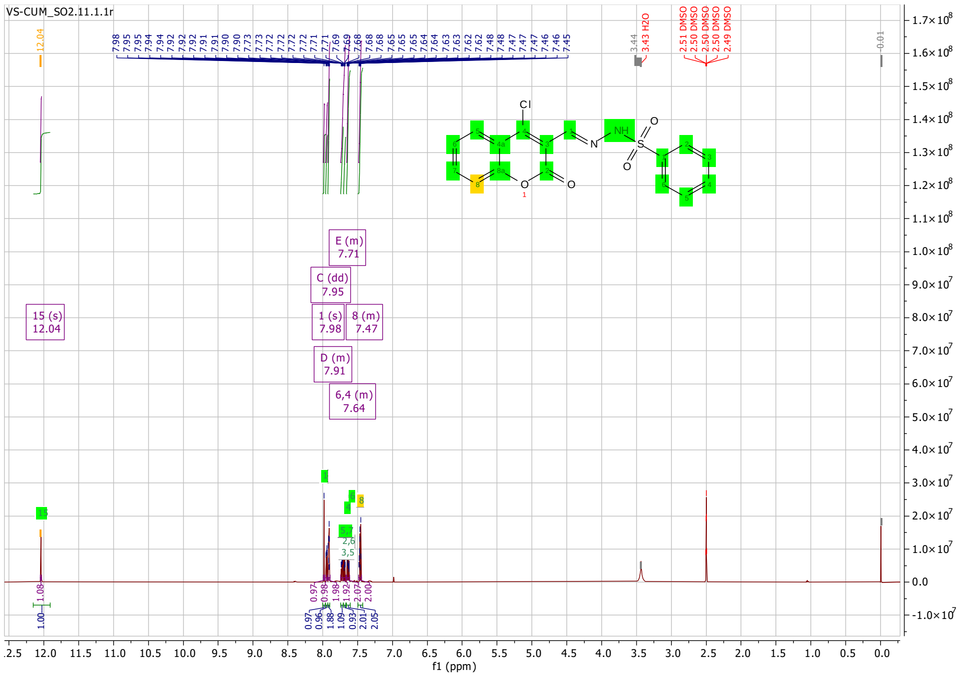

Figure S13. **^{1}H NMR spectrum of** ***N'-[(E)-(4-chloro-2-oxo-2H-1-benzopyran-3-yl)-methylidene] benzenesulfonohydrazide, 5a in DMSO-d6***

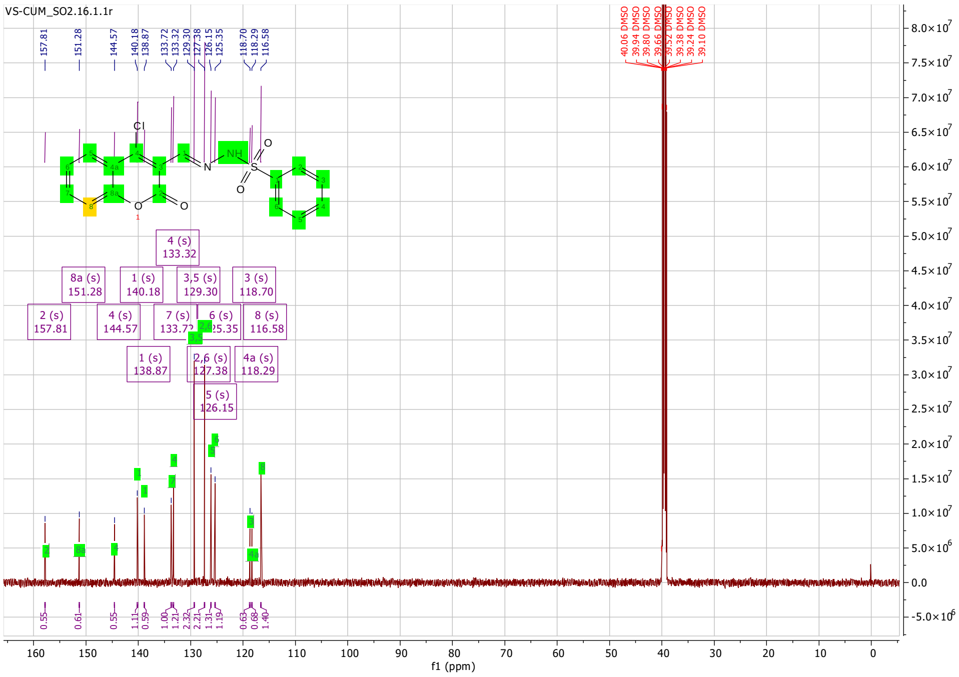

Figure S14. ^{13}**C NMR spectrum of** ***N'-[(E)-(4-chloro-2-oxo-2H-1-benzopyran-3-yl)-methylidene] benzenesulfonohydrazide, 5a in DMSO-d6***

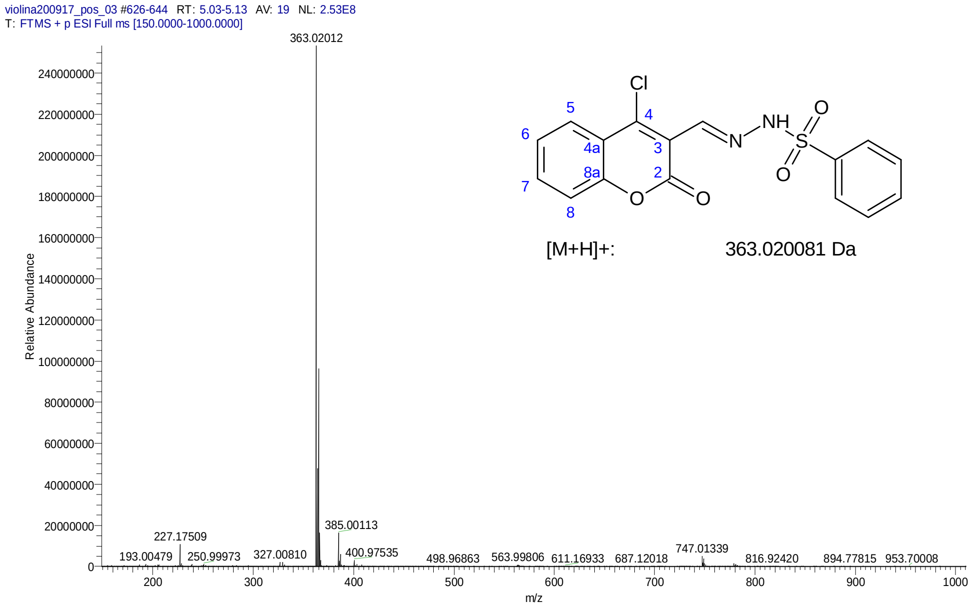

Figure S15. HRMS of ***N'-[(E)-(4-chloro-2-oxo-2H-1-benzopyran-3-yl)-methylidene] benzenesulfonohydrazide, 5a***

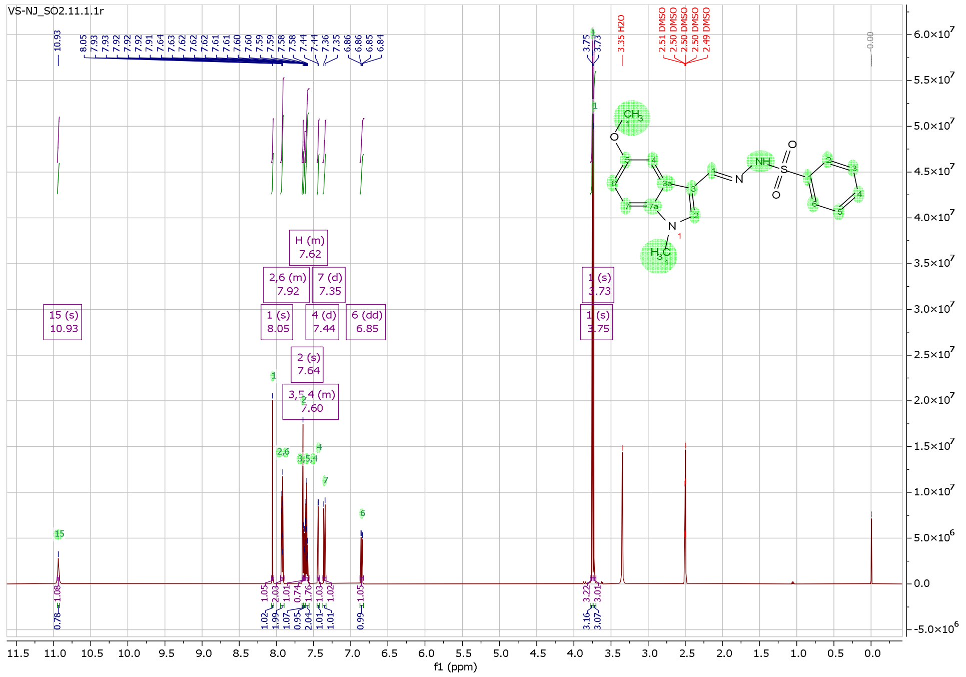

Figure S16. ^{1}**H NMR spectrum of** ***N'-[(E)-(5-methoxy-1-methyl-1H-indol-3-yl)methylidene]benzenesulfonohydrazide, 5b in DMSO-d6***

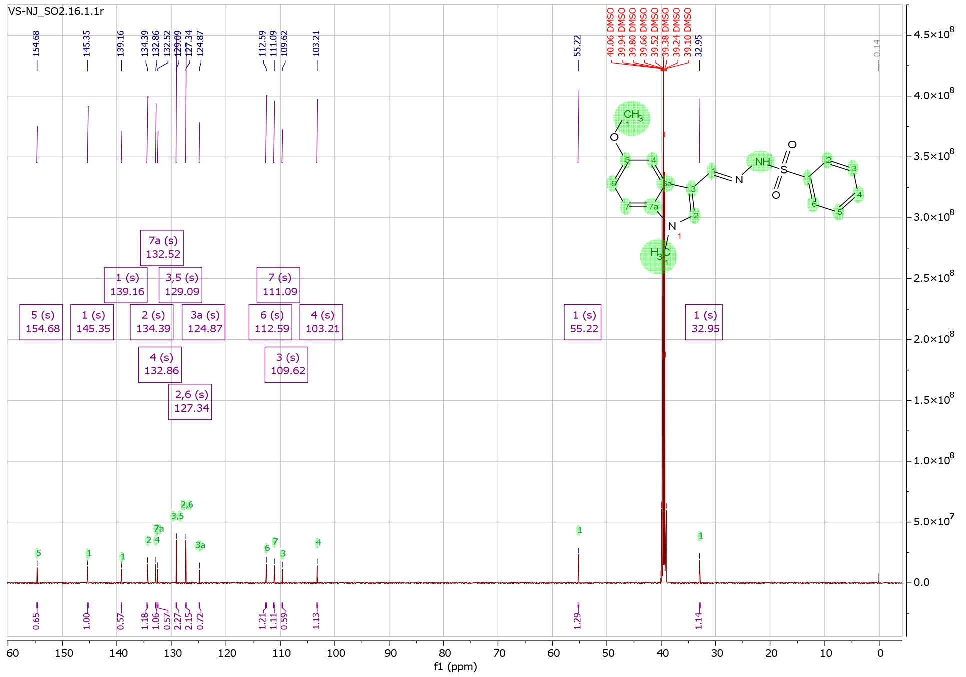

Figure S17. ^{13}**C NMR spectrum of** ***N'-[(E)-(5-methoxy-1-methyl-1H-indol-3-yl)methylidene]benzenesulfonohydrazide, 5b in DMSO-d6***

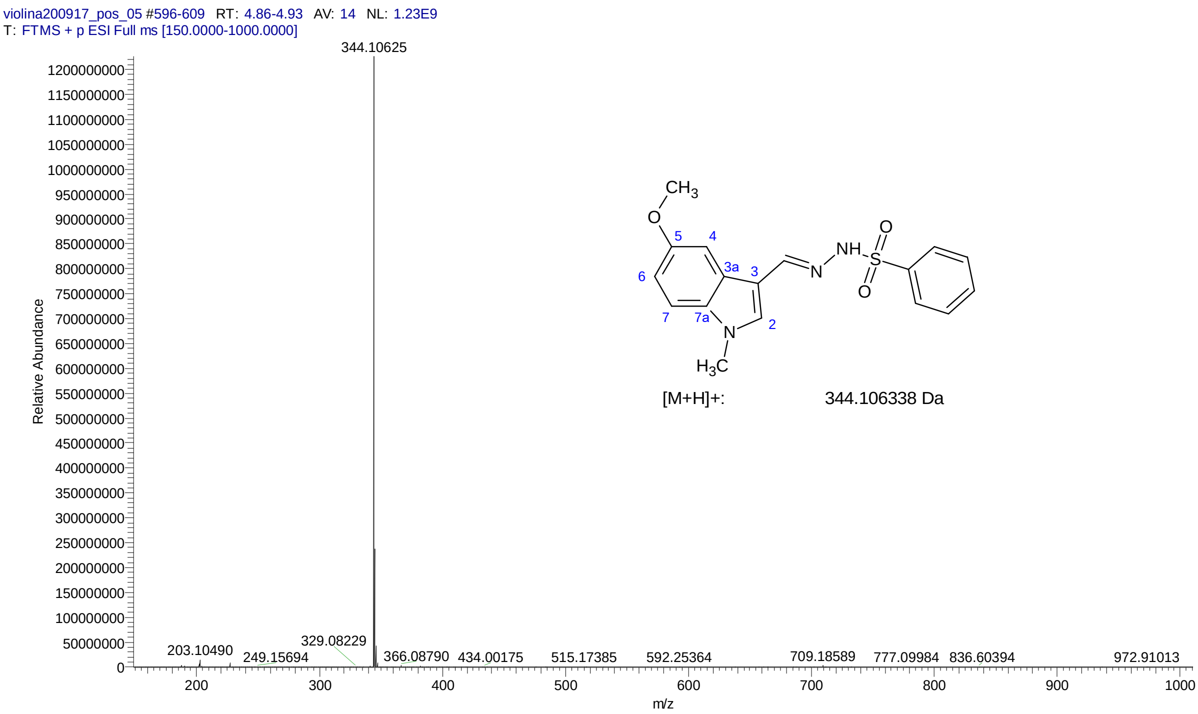

Figure S18. HRMS of *N'-[(E)-(5-methoxy-1-methyl-1H-indol-3-yl)methylidene]benzenesulfonohydrazide, 5b*

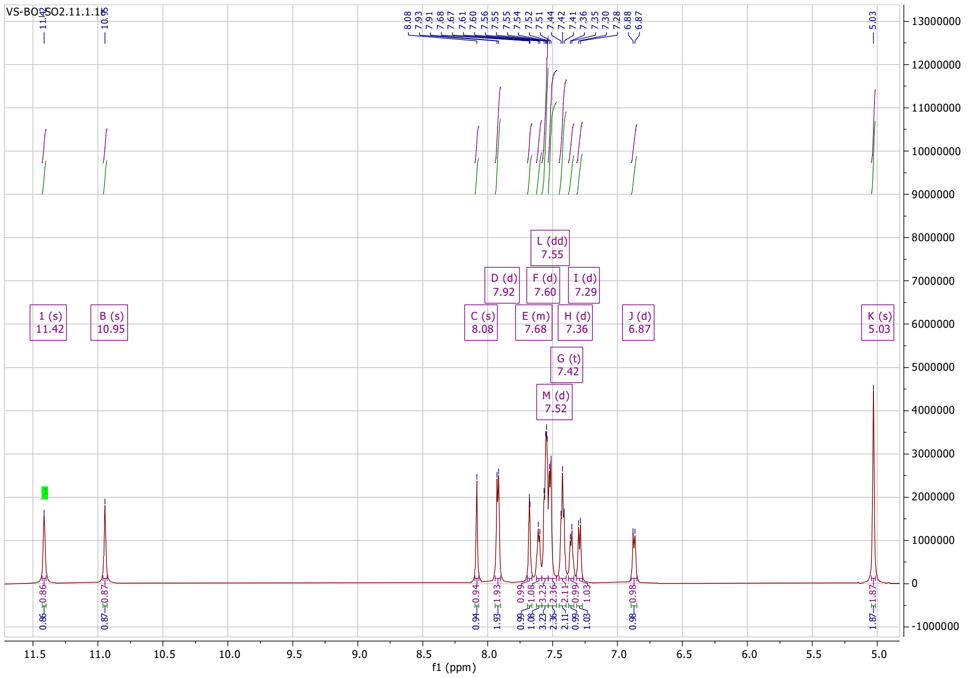

Figure S19. ^{1}H NMR spectrum of *N'-{(E)-[5-(benzyloxy)-1H-indol-3-yl]methylidene}benzenesulfonohydrazide, 5c in DMSO-d6*

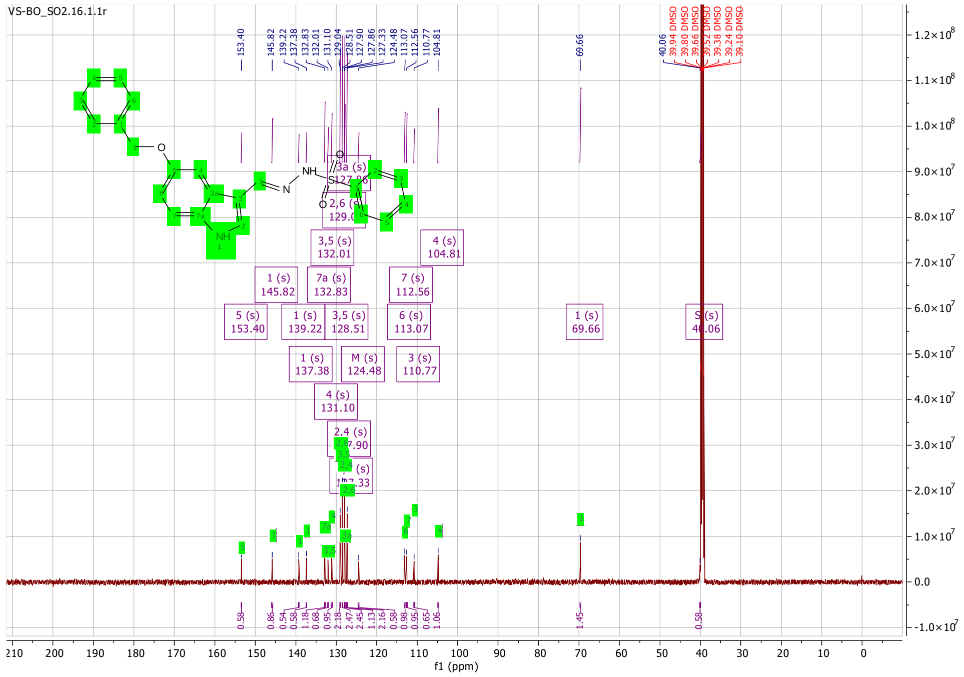

Figure S20. ^{13}C NMR spectrum of *N'-{(E)-[5-(benzyloxy)-1H-indol-3-yl]methylidene}benzenesulfonohydrazide, 5c in DMSO-d6*

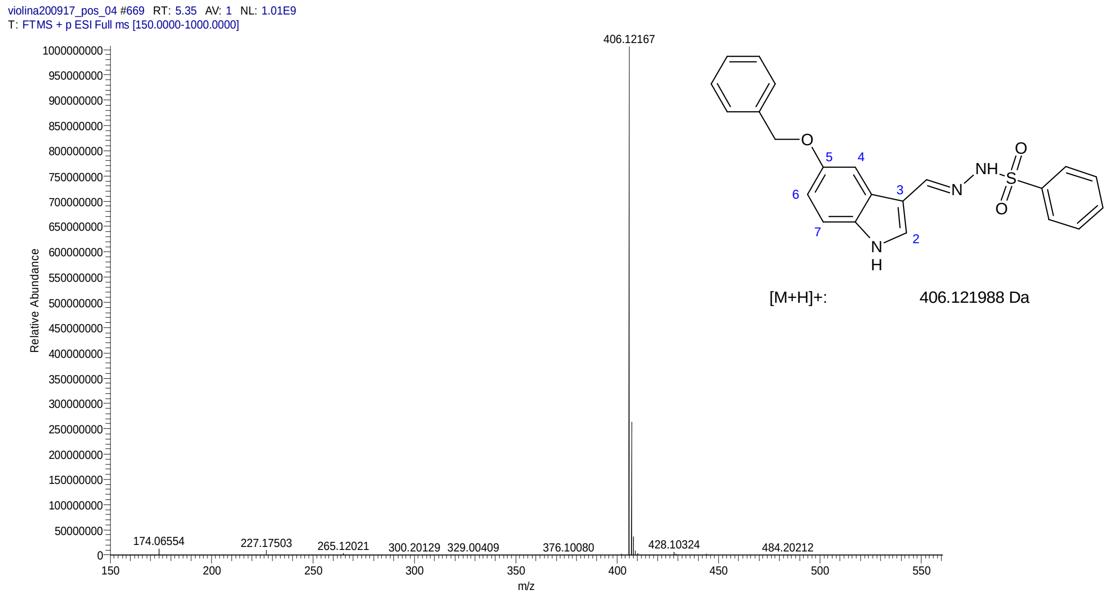

Figure S21. HRMS of *N'-{(E)-[5-(benzyloxy)-1H-indol-3-yl]methylidene}benzenesulfonohydrazide, 5c*

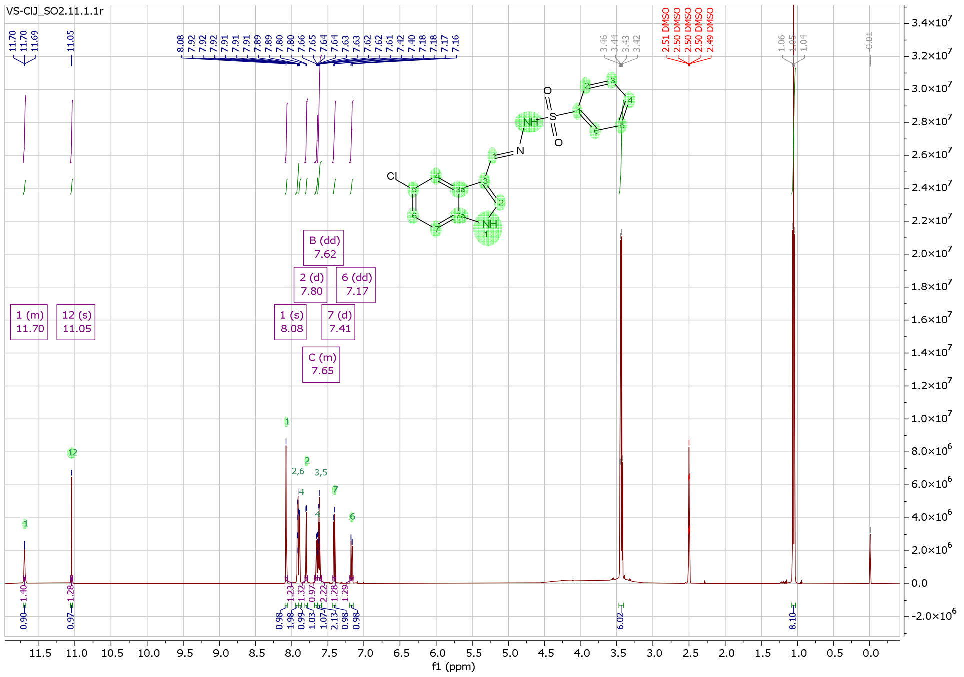

Figure S22. ^{1}**H NMR spectrum of** ***N'-[(E)-(5-chloro-1H-indol-3-yl)methylidene]benzenesulfonohydrazide, 5d in DMSO-d6***

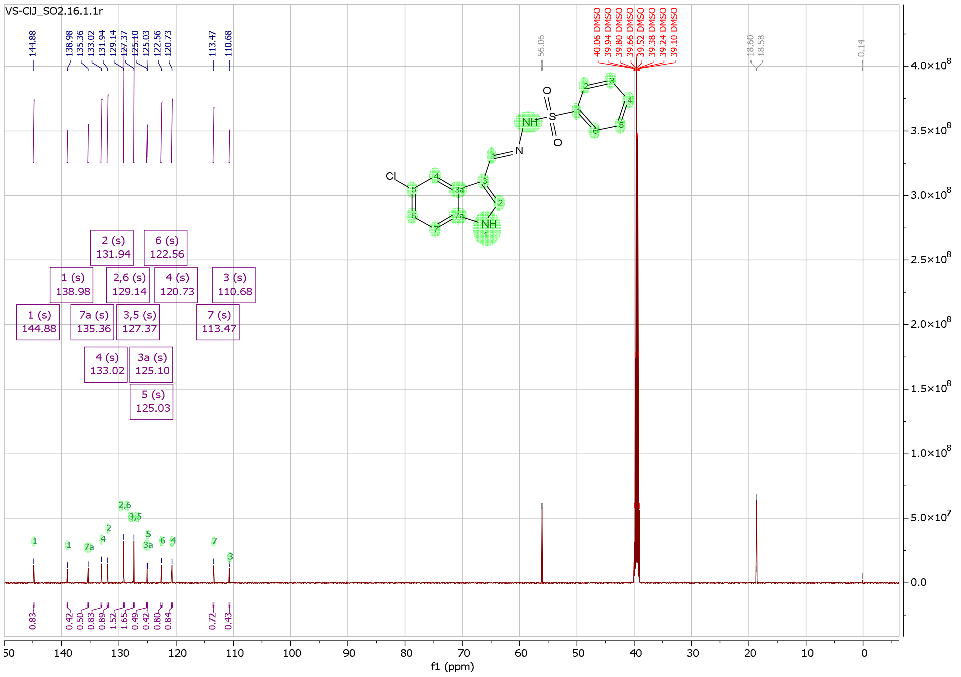

Figure S23. ^{13}**C NMR spectrum of** ***N'-[(E)-(5-chloro-1H-indol-3-yl)methylidene]benzenesulfonohydrazide, 5d in DMSO-d6***

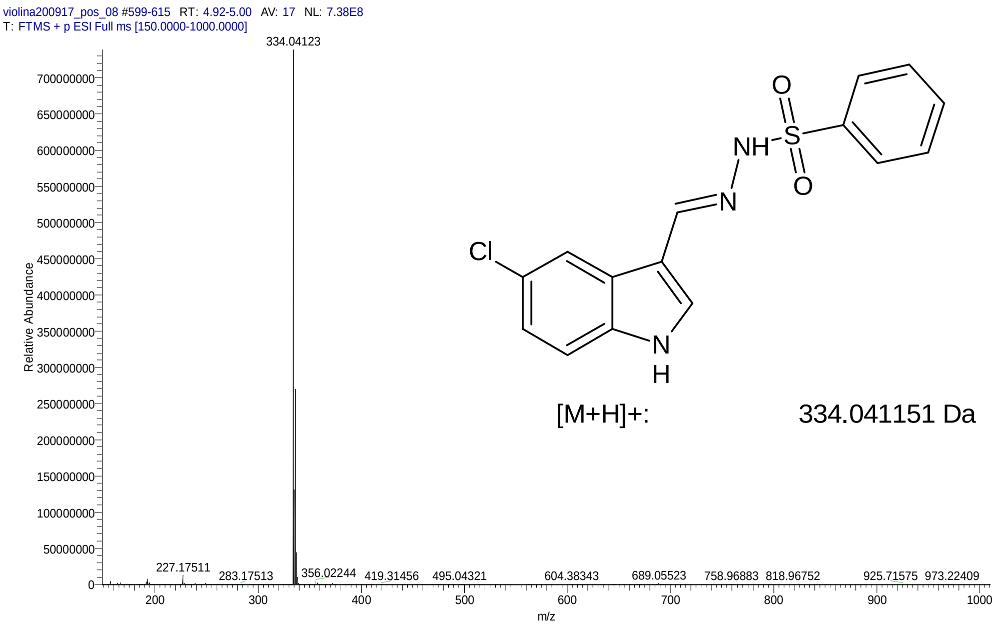

Figure S24. HRMS of *N'-[(Z)-(5-chloro-1H-indol-3-yl)methylidene]benzenesulfonohydrazide, 5d*

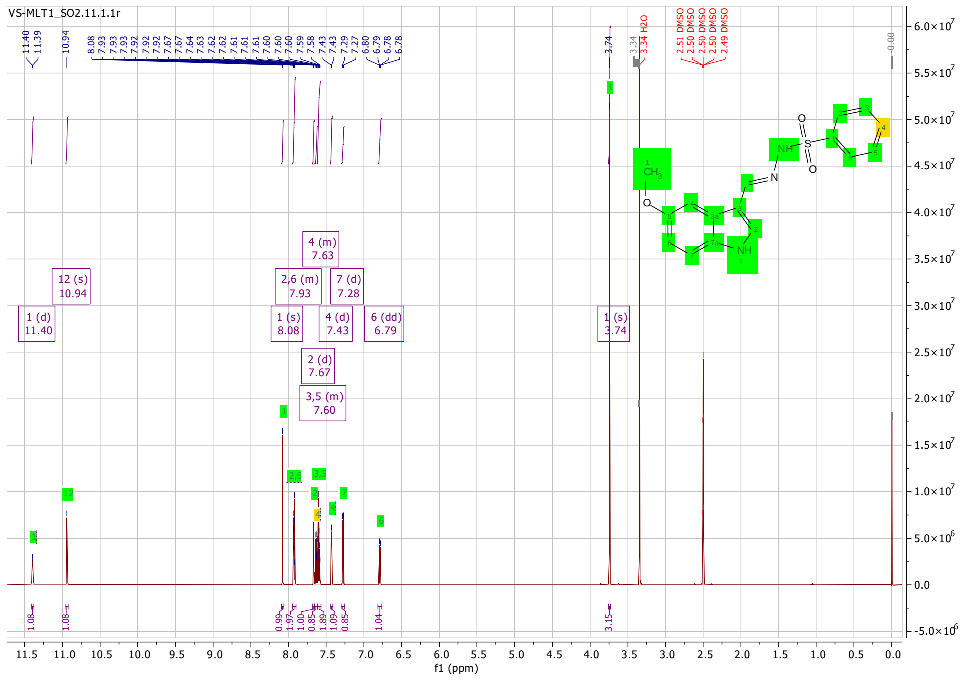

Figure S25. ¹H NMR spectrum of *N'-[(E)-(5-methoxy-1H-indol-3-yl)methylidene]benzenesulfonohydrazide, 5e in DMSO-d6*

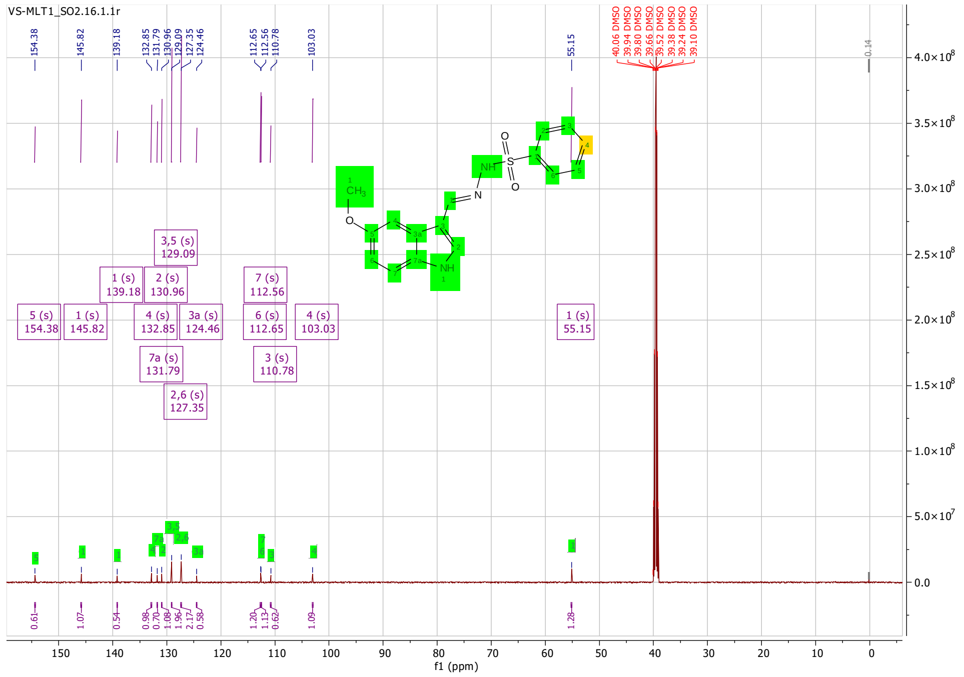

Figure S26. ^{13}**C NMR spectrum of** ***N'-[(E)-(5-methoxy-1H-indol-3-yl)methylidene]benzenesulfonohydrazide, 5e in DMSO-d6***

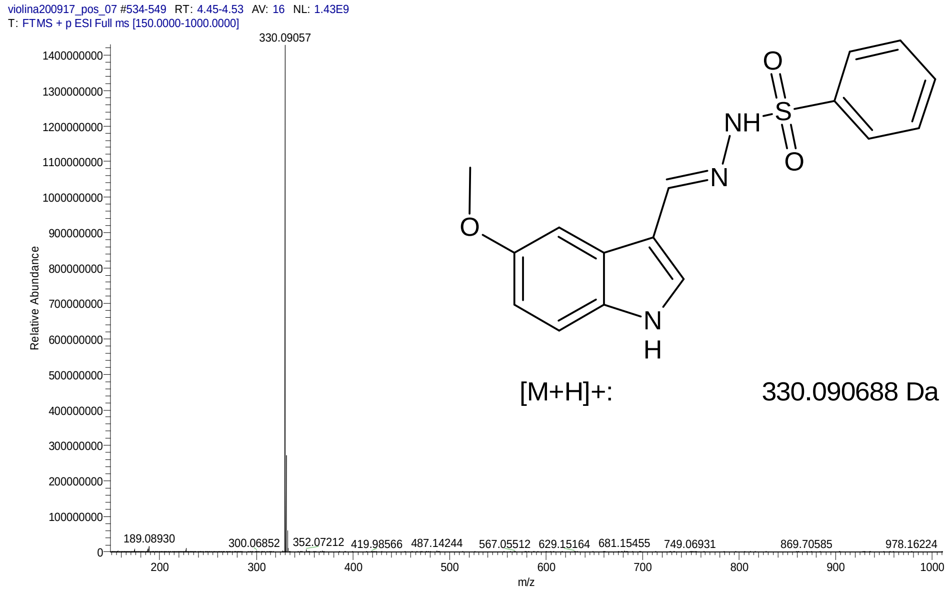

Figure S27. HRMS of ***N'-[(E)-(5-methoxy-1H-indol-3-yl)methylidene]benzenesulfonohydrazide, 5e***

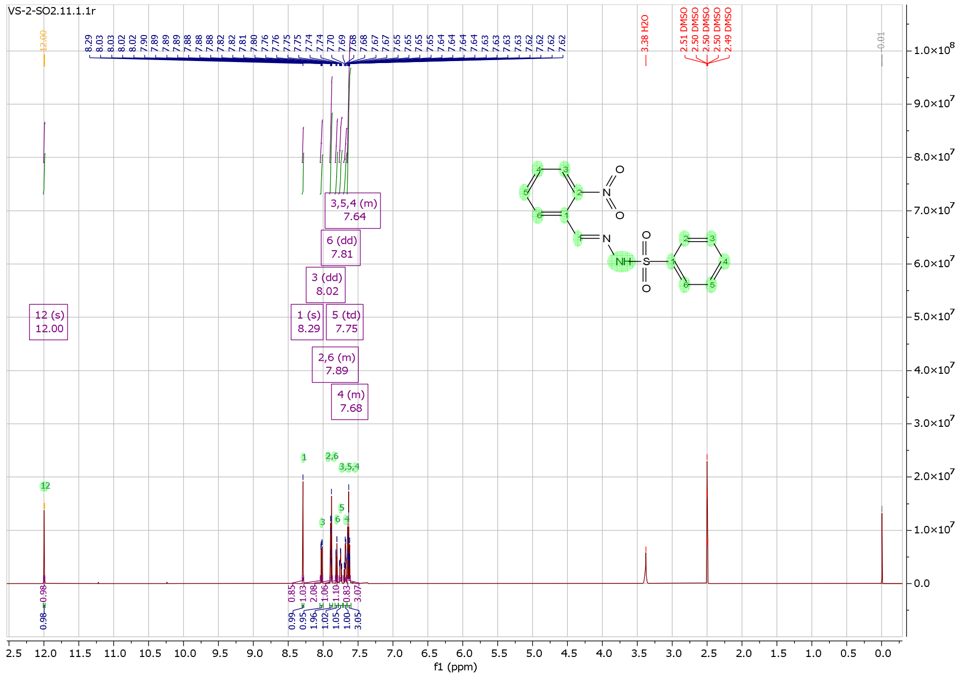

Figure S28. ^{1}H **NMR spectrum of** ***N'-[(E)-(2-nitrophenyl)methylidene]benzenesulfonohydrazide, 5f in DMSO-d6***

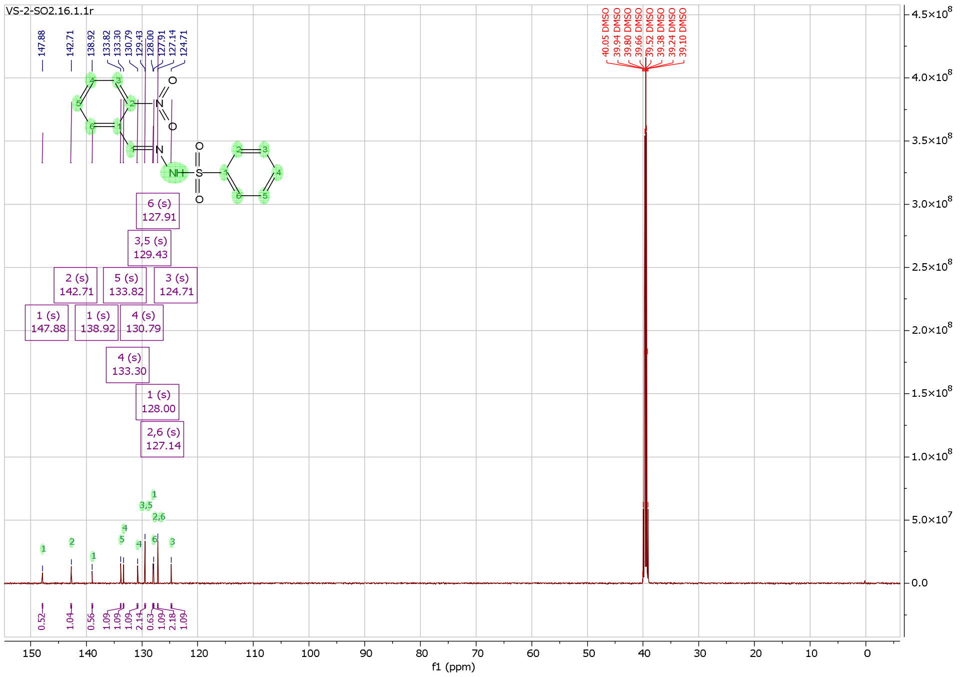

Figure S29. ^{13}C **NMR spectrum of** ***N'-[(E)-(2-nitrophenyl)methylidene]benzenesulfonohydrazide, 5f in DMSO-d6***

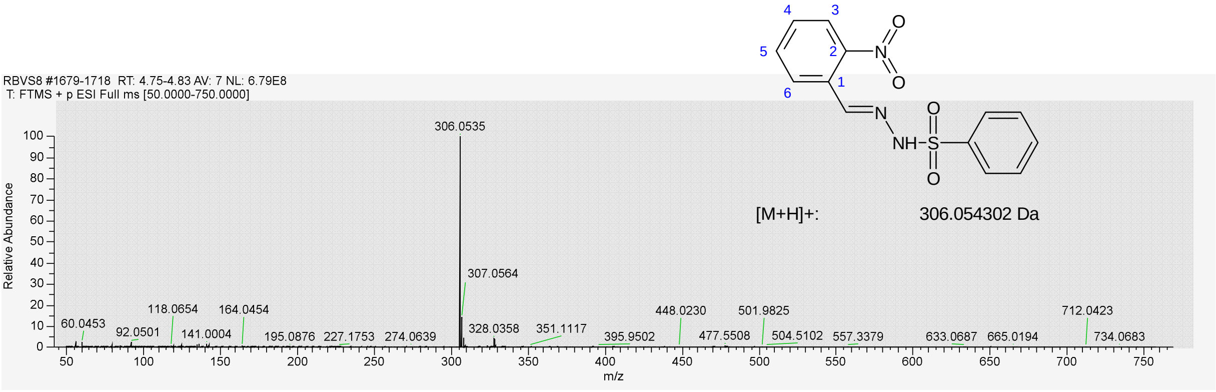

Figure S30. HRMS of *N'-[(E)-(2-nitrophenyl)methylidene]benzenesulfonohydrazide, 5f*

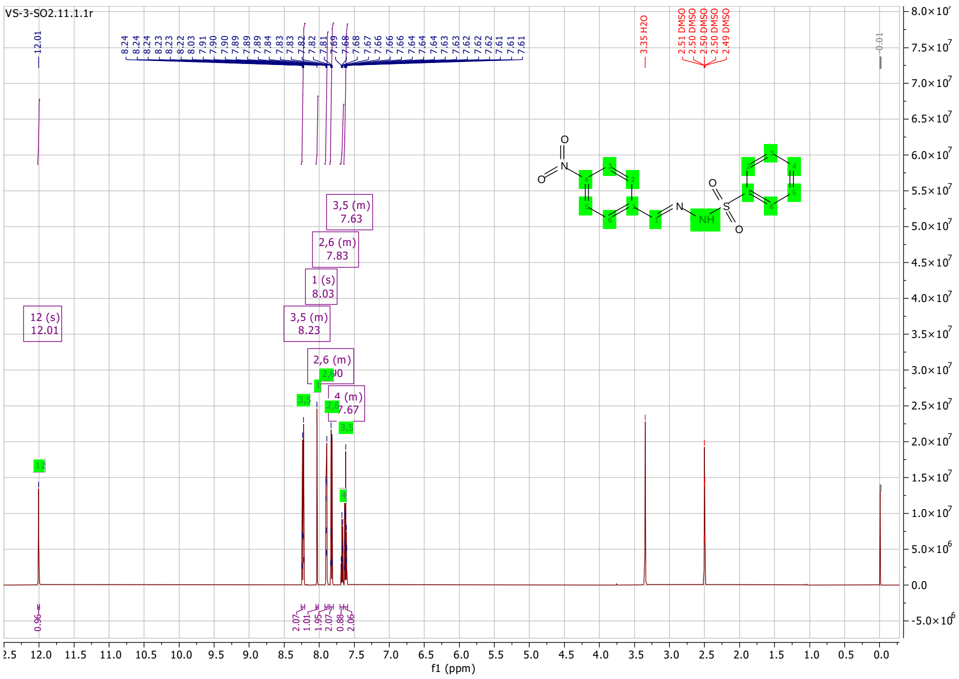

Figure S31. ^{1}H NMR spectrum of *N'-[(E)-(4-nitrophenyl)methylidene]benzenesulfonohydrazide, 5g in DMSO-d6*

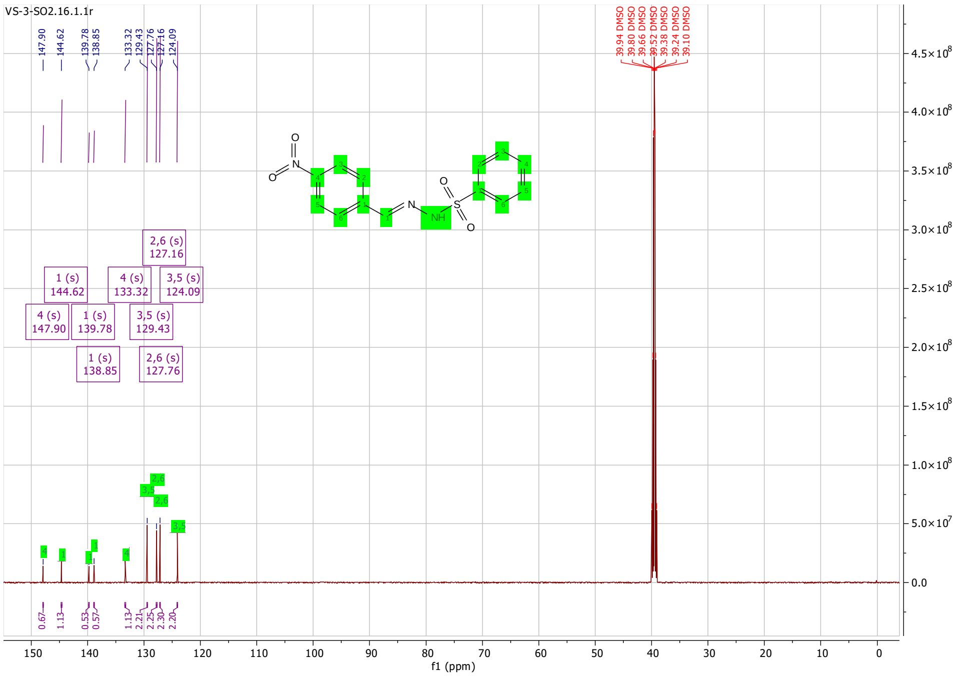

Figure S32. ^{13}**C NMR spectrum of** ***N'-[(E)-(4-nitrophenyl)methylidene]benzenesulfonohydrazide, 5g in DMSO-d6***

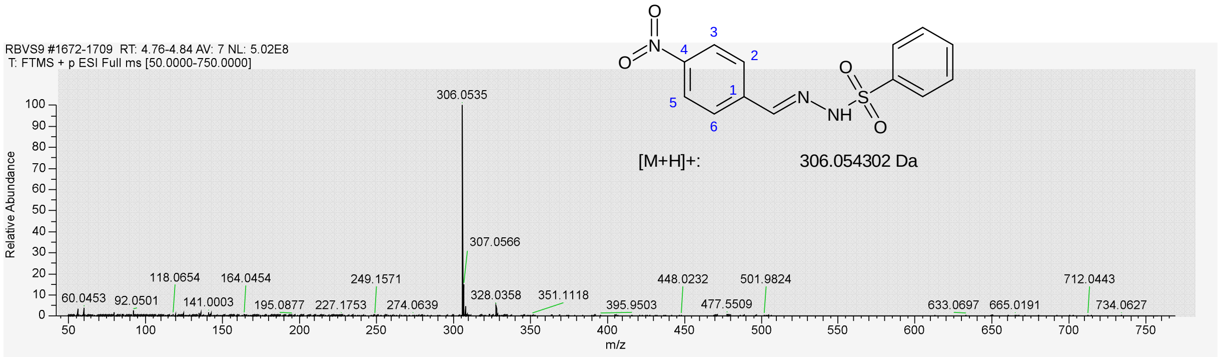

Figure S33. HRMS of ***N'-[(E)-(4-nitrophenyl)methylidene]benzenesulfonohydrazide, 5g***

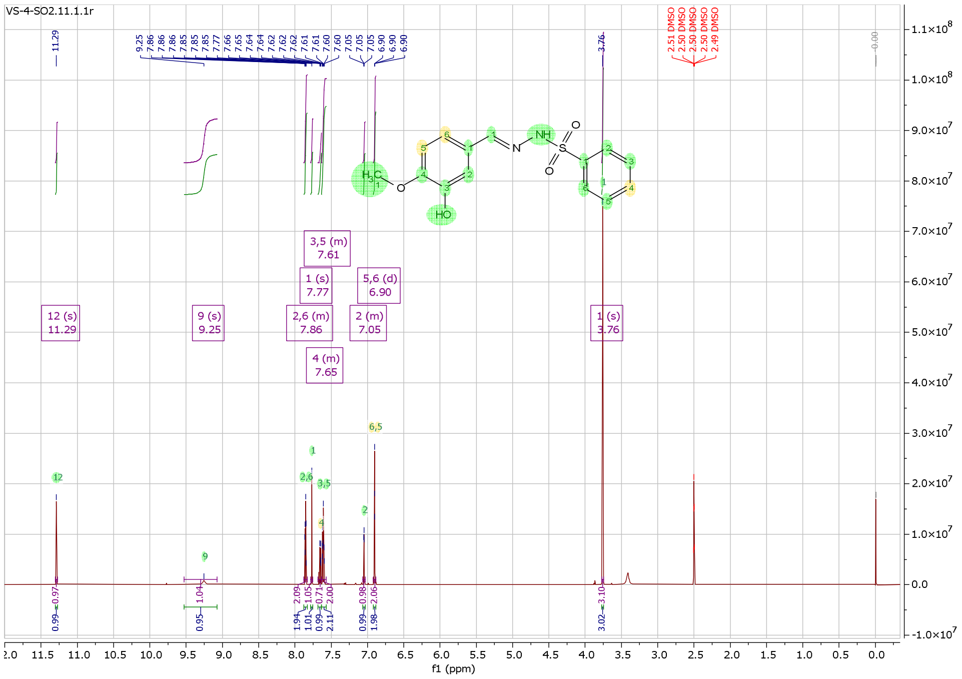

Figure S34. ^{1}H **NMR spectrum of** ***N'-[(E)-(3-hydroxy-4-methoxyphenyl)methylidene]benzenesulfonohydrazide, 5h in DMSO-d6***

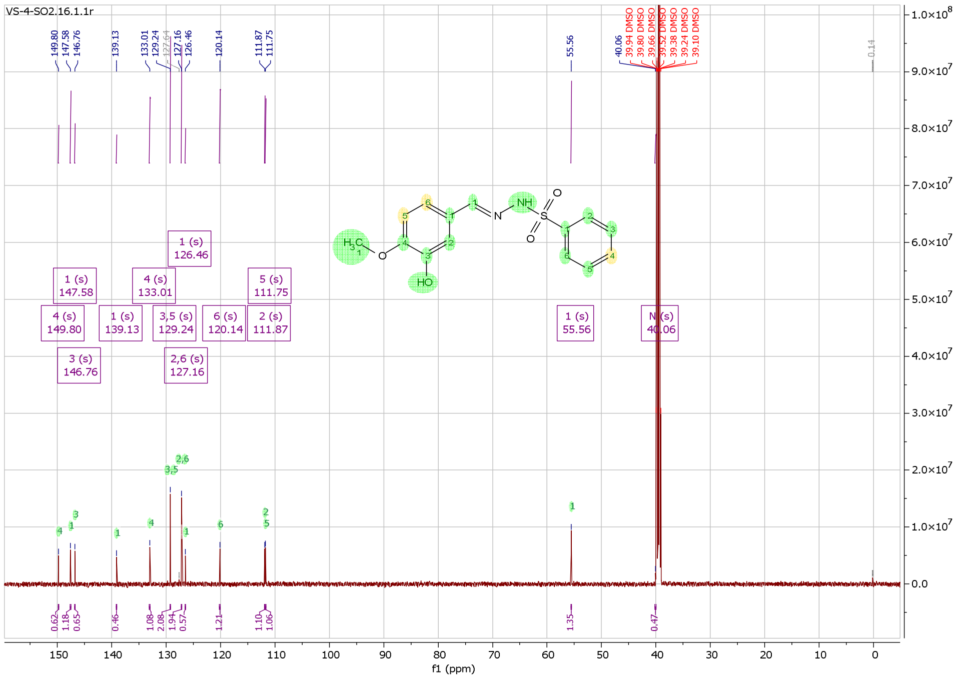

Figure S35. ^{13}C **NMR spectrum of** ***N'-[(E)-(3-hydroxy-4-methoxyphenyl)methylidene]benzenesulfonohydrazide, 5h in DMSO-d6***

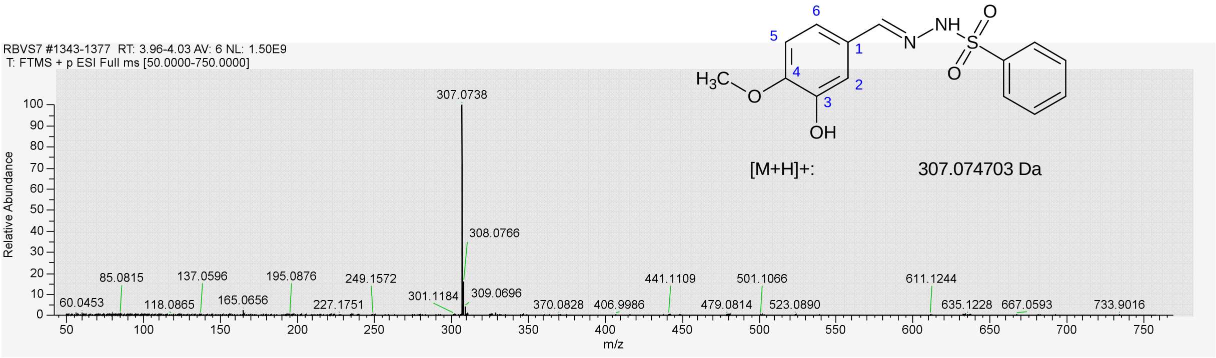

Figure S36. HRMS of *N'-[(E)-(3-hydroxy-4-methoxyphenyl)methylidene]benzenesulfonohydrazide, 5h*

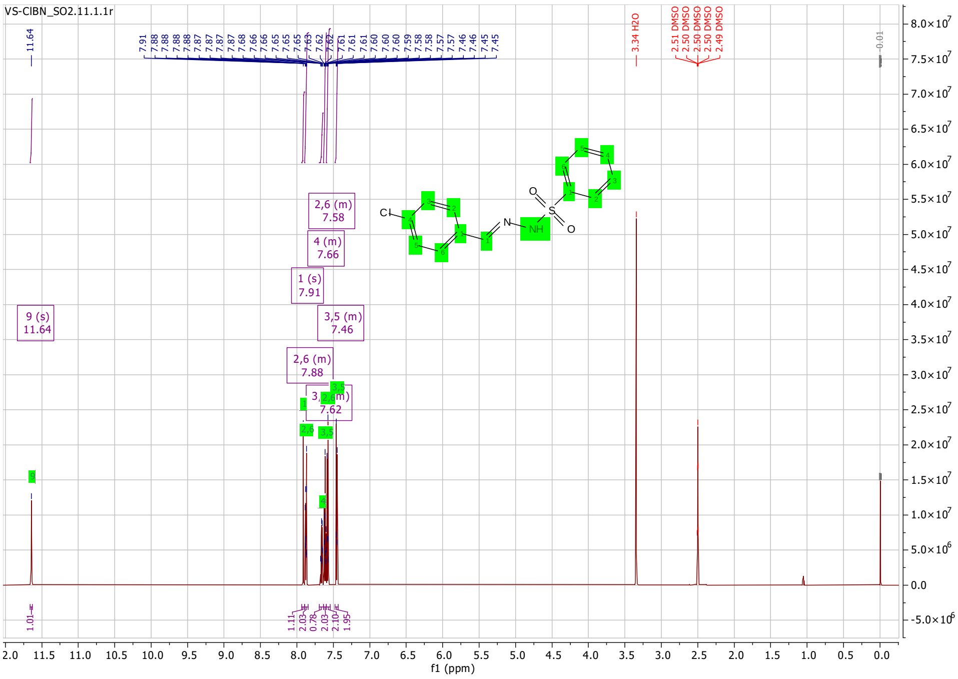

Figure S37. ^{1}H NMR spectrum of *N'-[(E)-(4-chlorophenyl)methylidene]benzenesulfonohydrazide, 5i in DMSO-d6*

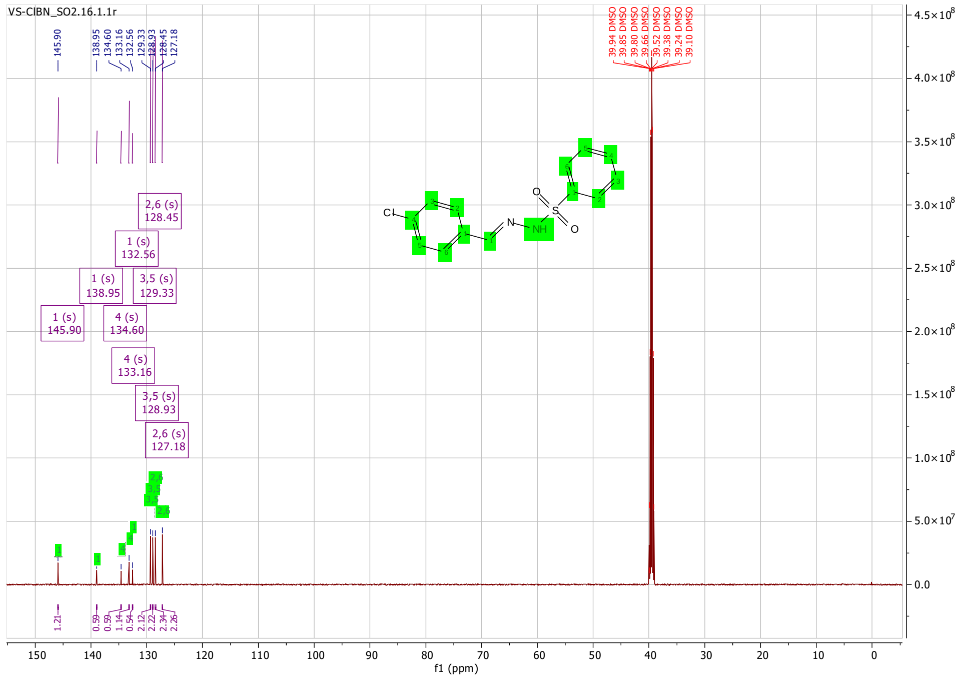

Figure S38. ^{13}**C NMR spectrum of** ***N'-[(E)-(4-chlorophenyl)methylidene]benzenesulfonohydrazide, 5i in DMSO-d6***

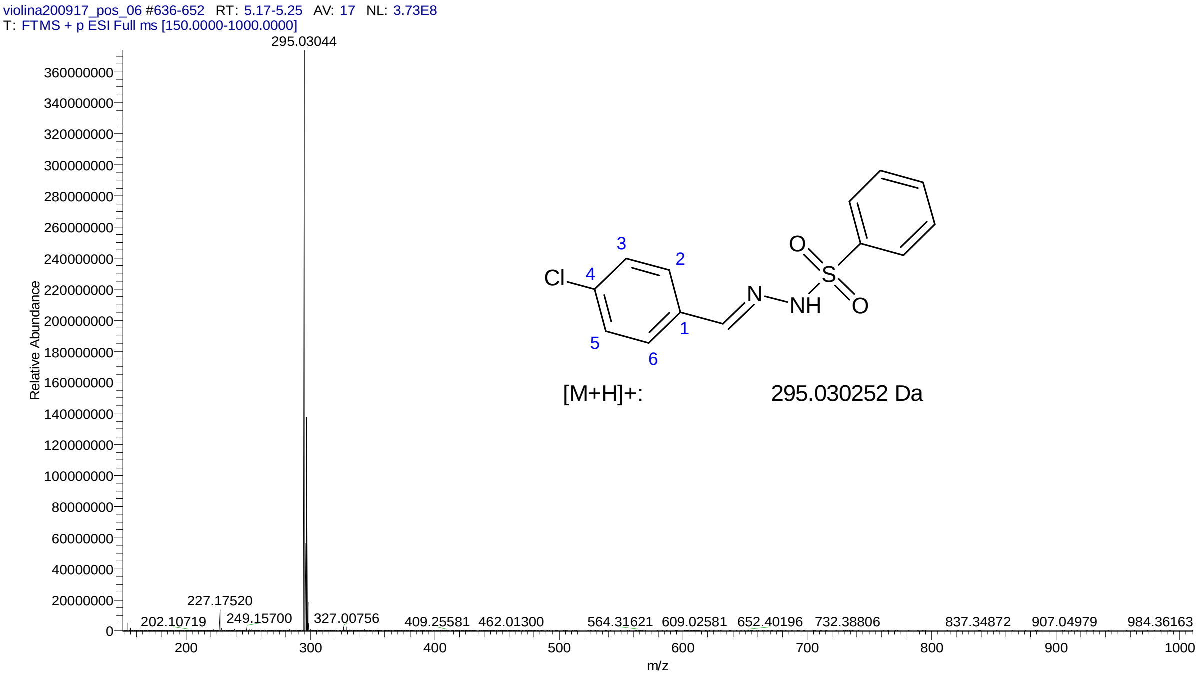

Figure S39. HRMS of ***N'-[(E)-(4-chlorophenyl)methylidene]benzenesulfonohydrazide, 5i***

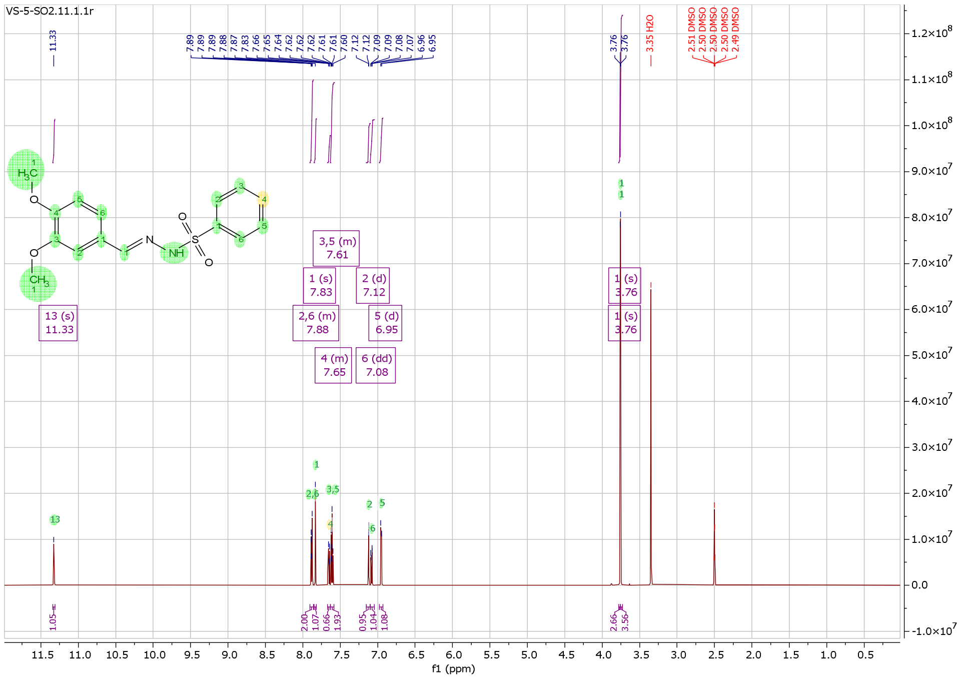

Figure S40. [1]**H NMR spectrum of** ***N'-[(E)-(3,4-dimethoxyphenyl)methylidene]benzenesulfonohydrazide, 5j in DMSO-d6***

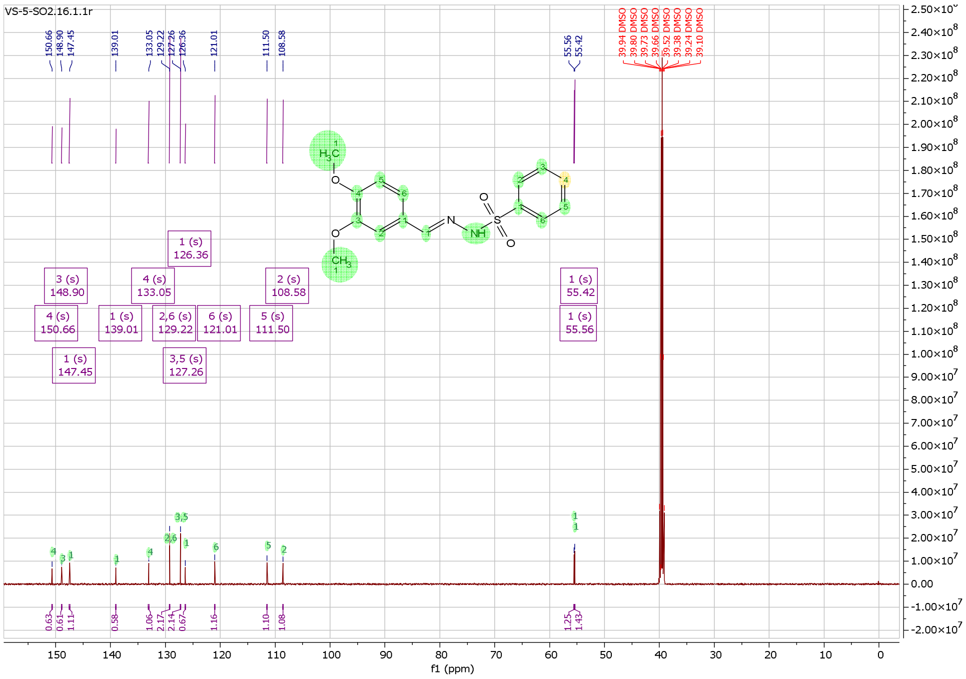

Figure S41. [13]**C NMR spectrum of** ***N'-[(E)-(3,4-dimethoxyphenyl)methylidene]benzenesulfonohydrazide, 5j in DMSO-d6***

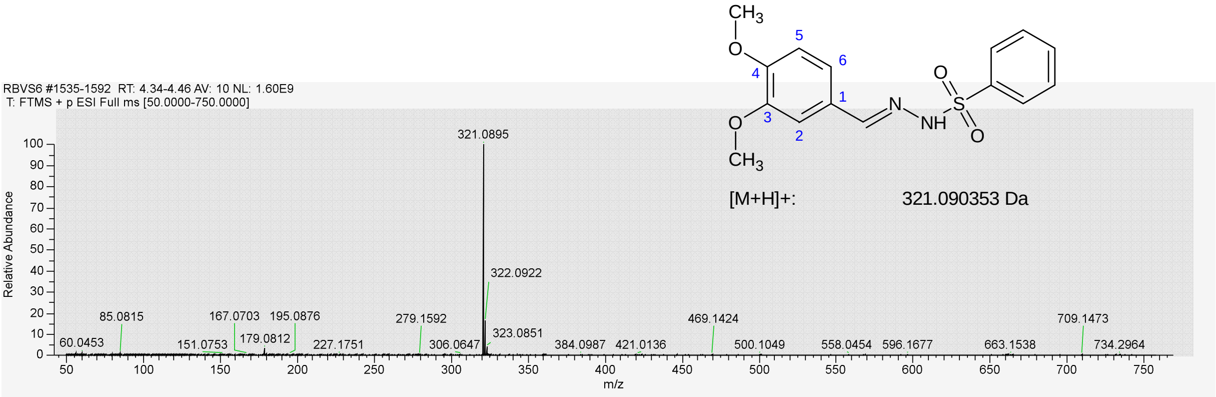

Figure S42. HRMS of *N'-[(E)-(3,4-dimethoxyphenyl)methylidene]benzenesulfonohydrazide, 5j*

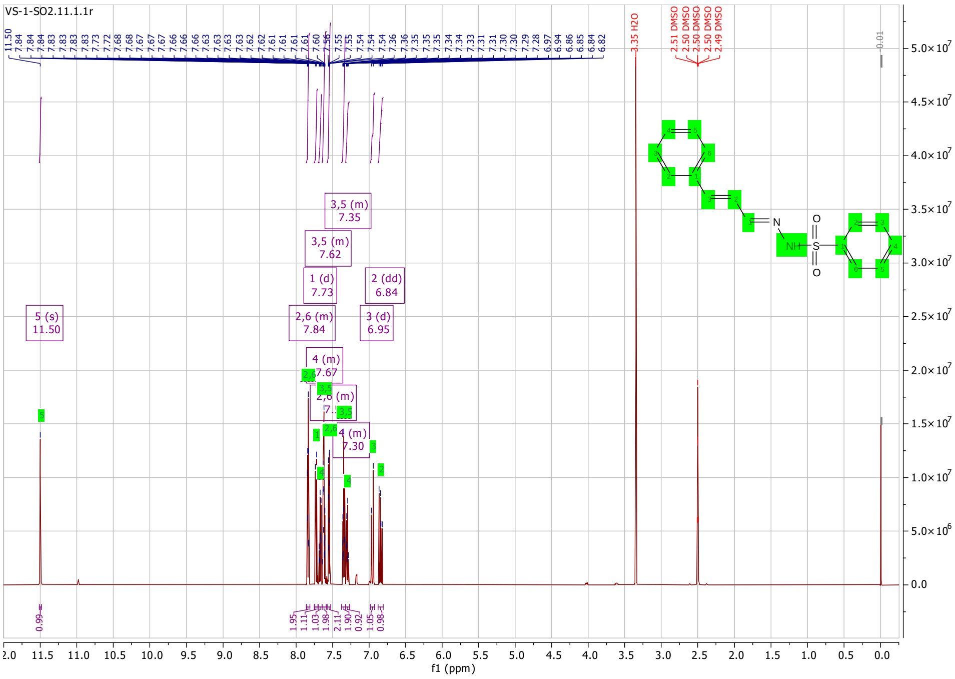

Figure S43. ^{1}H NMR spectrum of *N'-[(1E,2E)-3-phenylprop-2-en-1-ylidene]benzenesulfonohydrazide, 5k in DMSO-d6*

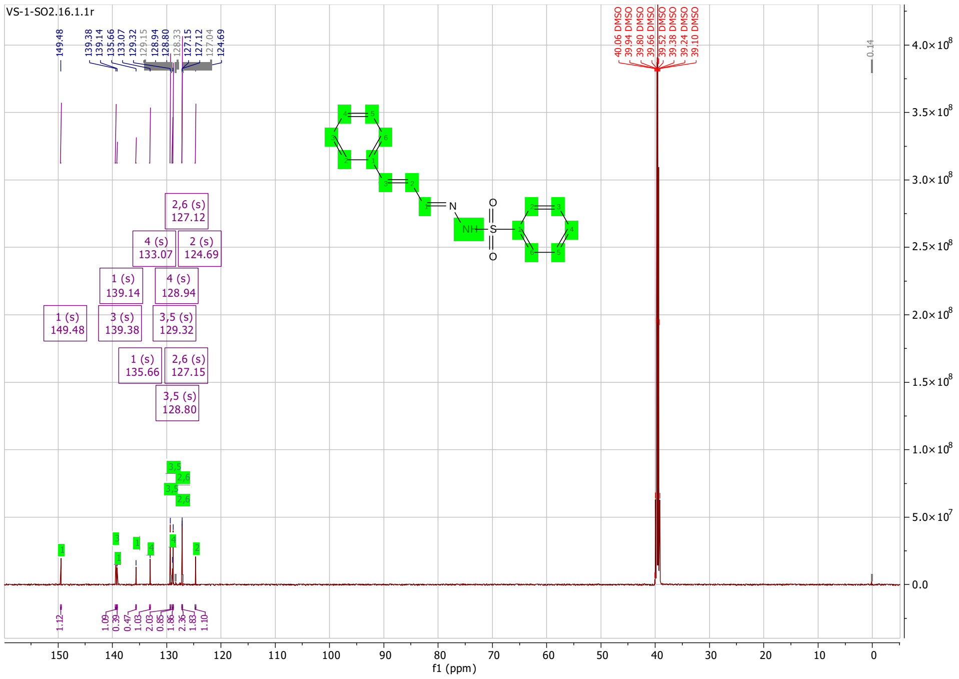

Figure S44. ^{13}**C NMR spectrum of** ***N'-[(1E,2E)-3-phenylprop-2-en-1-ylidene]benzenesulfonohydrazide, 5k in DMSO-d6***

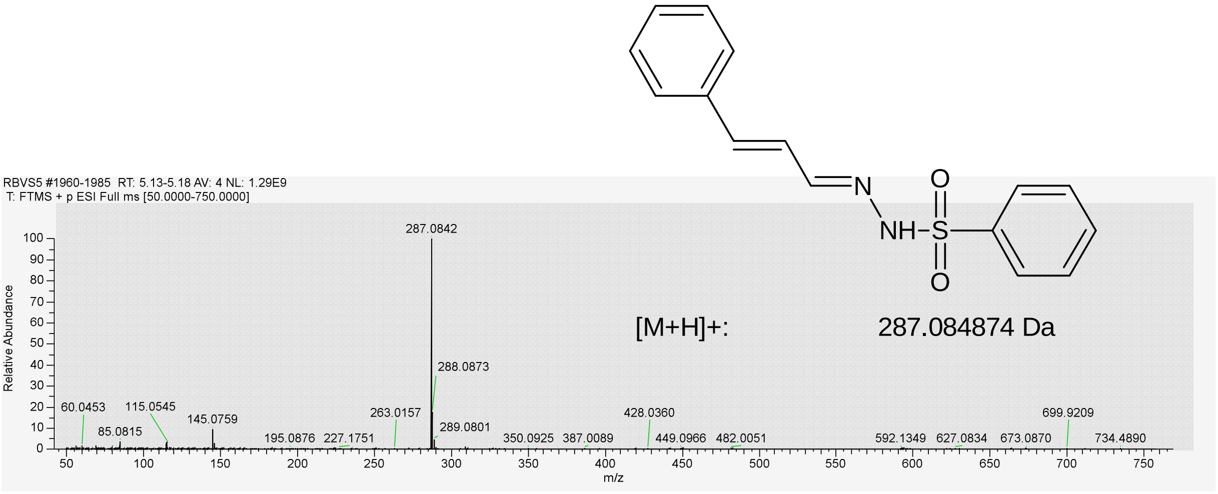

Figure S45. HRMS of ***N'-[(1E,2E)-3-phenylprop-2-en-1-ylidene]benzenesulfonohydrazide, 5k***
